# Supplementary material for: Human Impact on the Twenty-Four-Hour Patterns of Steller Sea Lions’ Use of a Haulout in Hokkaido, Japan
Source: Animals (Basel). 2024 Apr 27;14(9):1312. doi: 10.3390/ani14091312 (PMC11083395; doi:10.3390/ani14091312)
Supplement: Supplementary file 1 [file animals-14-01312-s001.zip › Figure S1_201701-201703.pdf]

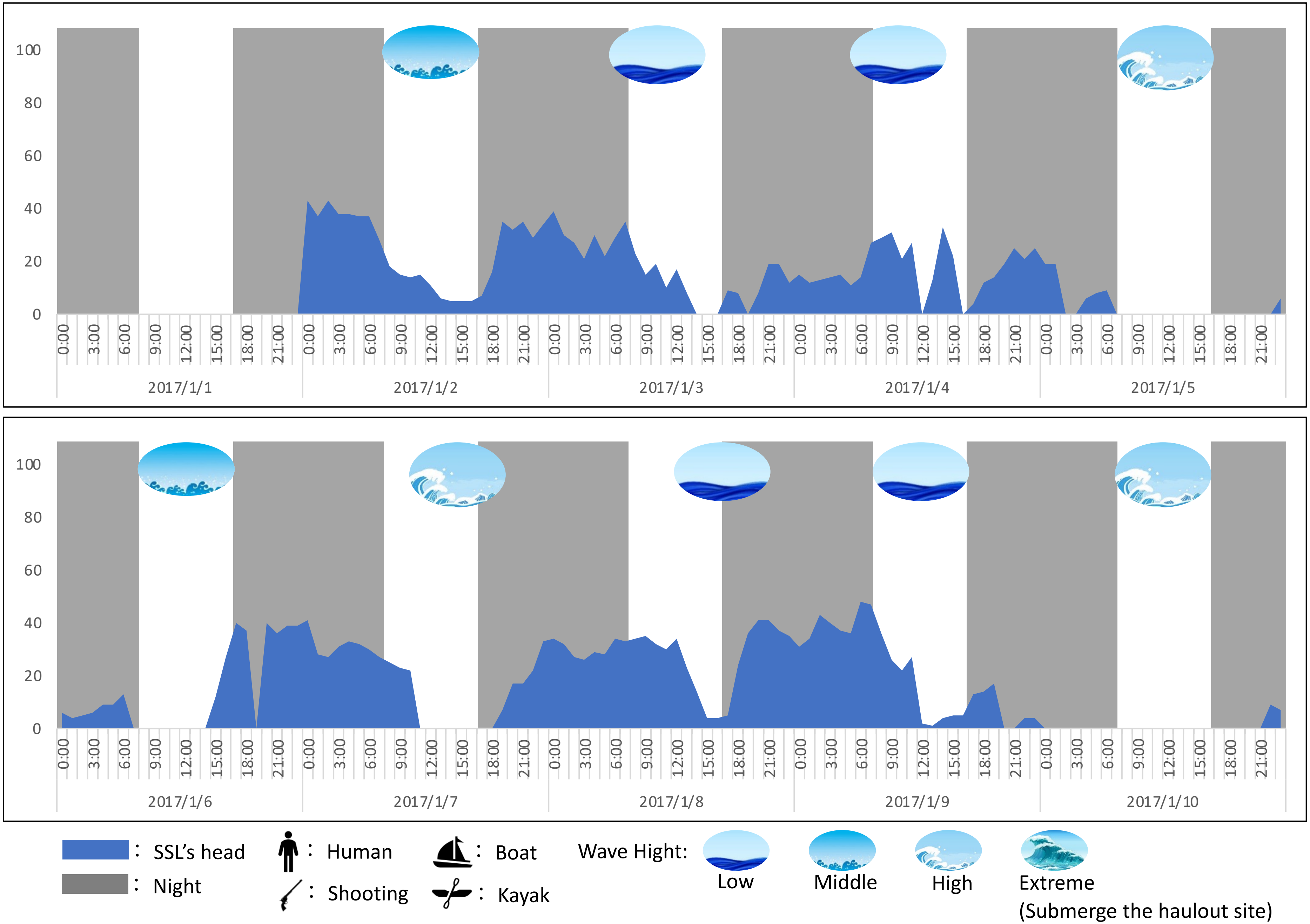

100
80
60
40
20
0
0:00
3:00
6:00
9:00
12:00
15:00
18:00
21:00
2017/1/1
2017/1/2
2017/1/3
2017/1/4
2017/1/5
2017/1/6
2017/1/7
2017/1/8
2017/1/9
2017/1/10
: SSL's head
: Night
: Human
: Shooting
: Boat
: Kayak
Wave Hight:
Low
Middle
High
Extreme
(Submerge the haulout site)

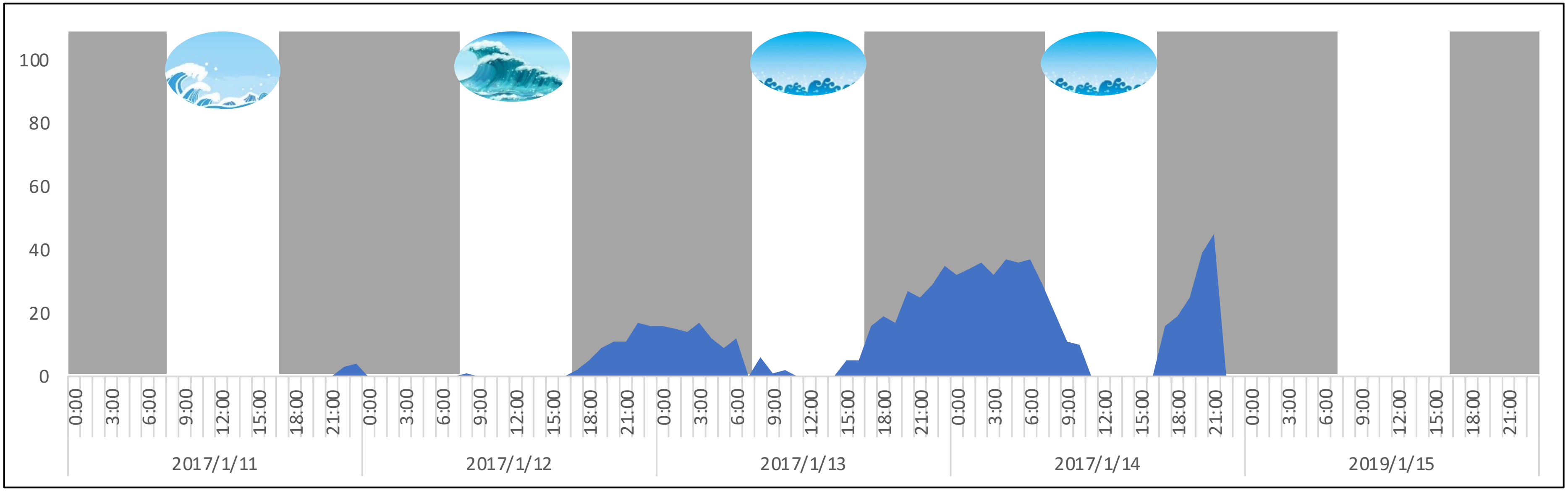

100
80
60
40
20
0
0:00 3:00 6:00 9:00 12:00 15:00 18:00 21:00
2017/1/11
2017/1/12
2017/1/13
2017/1/14
2019/1/15

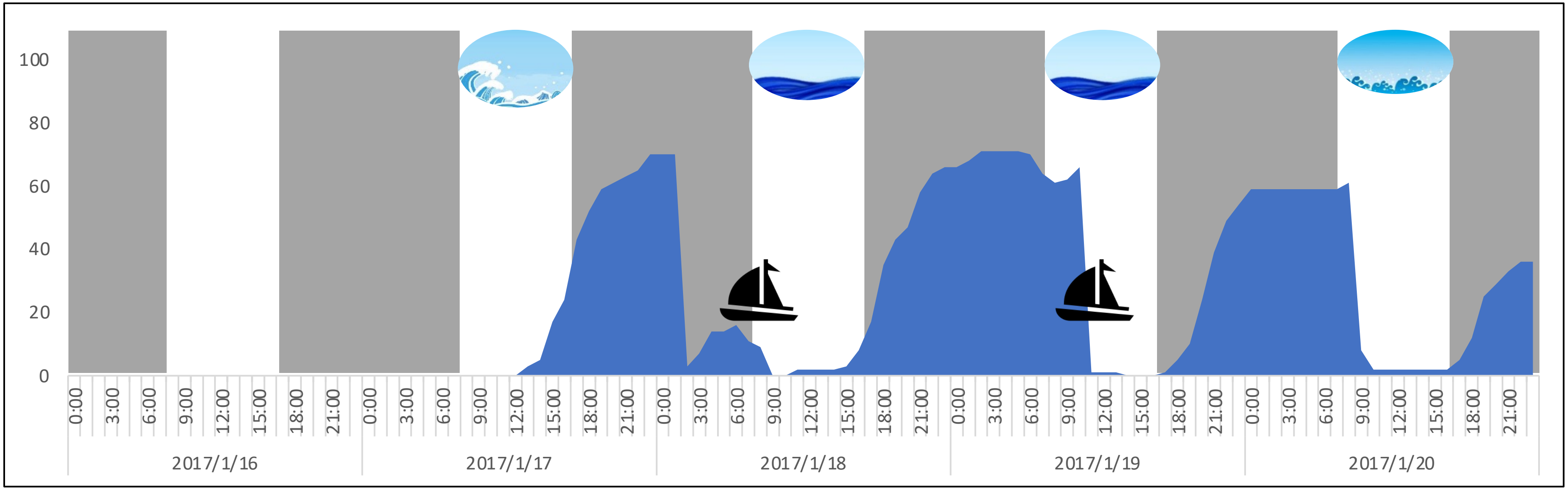

100
80
60
40
20
0
0:00 3:00 6:00 9:00 12:00 15:00 18:00 21:00
2017/1/16
2017/1/17
2017/1/18
2017/1/19
2017/1/20

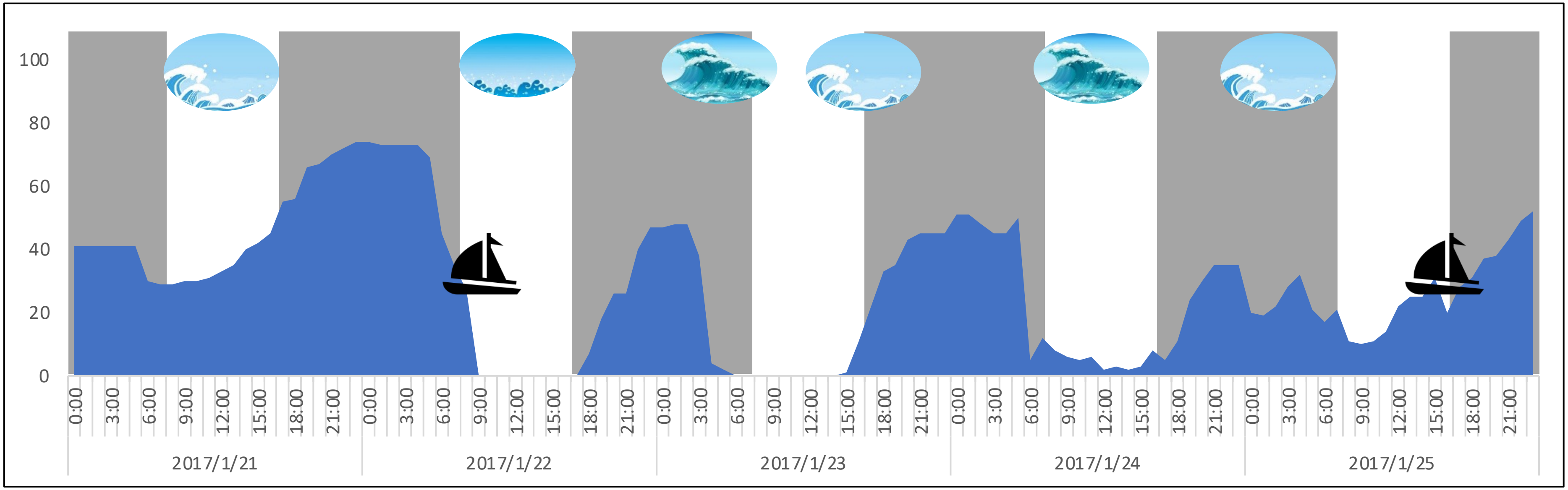

100
80
60
40
20
0
0:00 3:00 6:00 9:00 12:00 15:00 18:00 21:00
2017/1/21
2017/1/22
2017/1/23
2017/1/24
2017/1/25

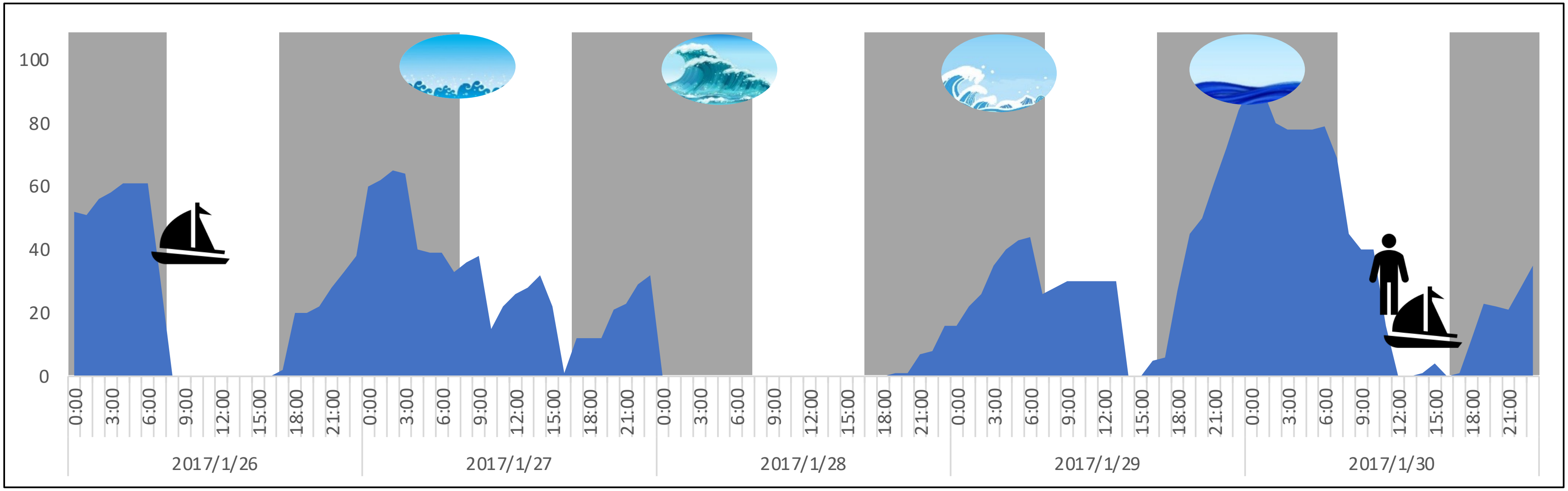

100
80
60
40
20
0
0:00 3:00 6:00 9:00 12:00 15:00 18:00 21:00
2017/1/26
2017/1/27
2017/1/28
2017/1/29
2017/1/30

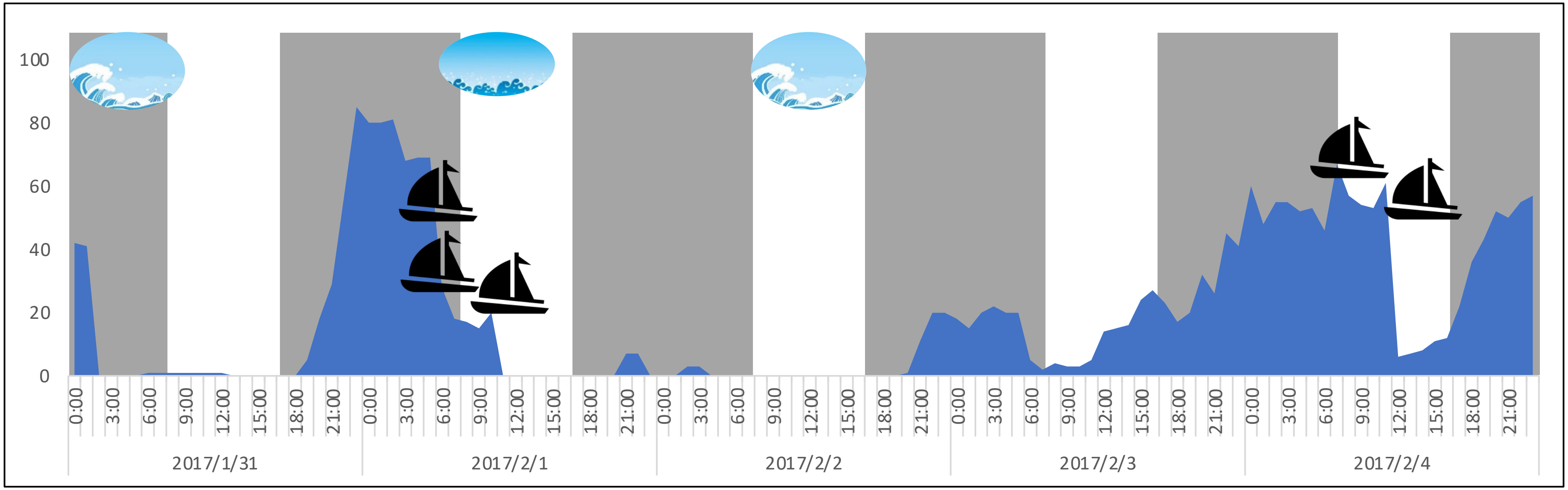
100
80
60
40
20
0
0:00
3:00
6:00
9:00
12:00
15:00
18:00
21:00
2017/1/31
2017/2/1
2017/2/2
2017/2/3
2017/2/4

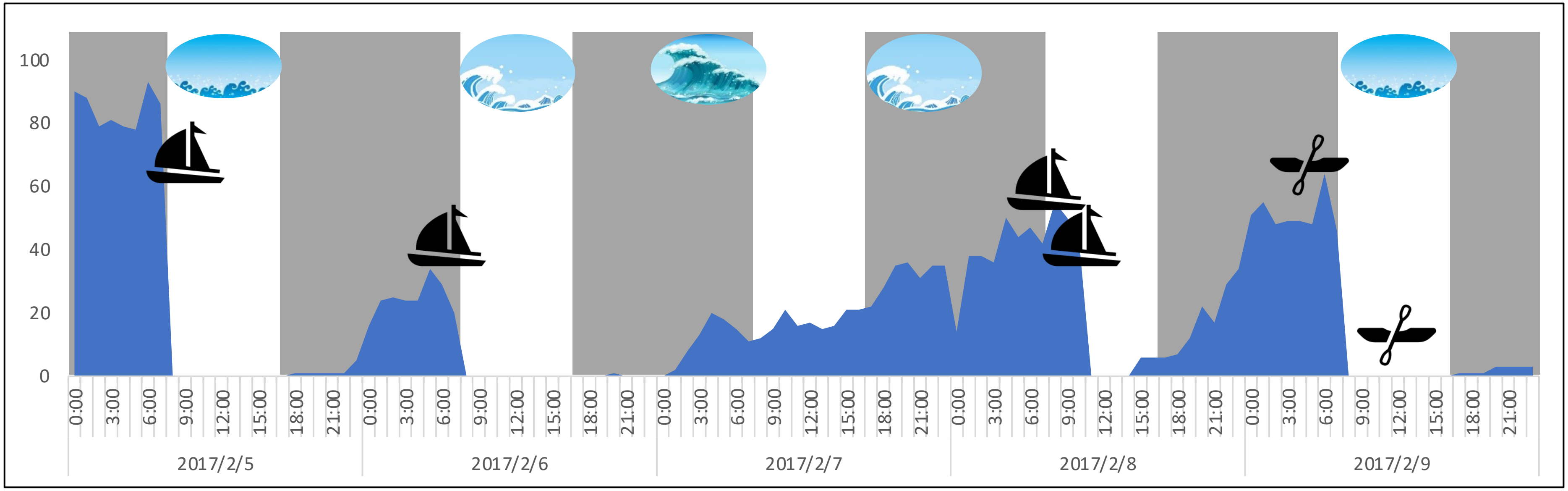
100
80
60
40
20
0
0:00
3:00
6:00
9:00
12:00
15:00
18:00
21:00
2017/2/5
2017/2/6
2017/2/7
2017/2/8
2017/2/9

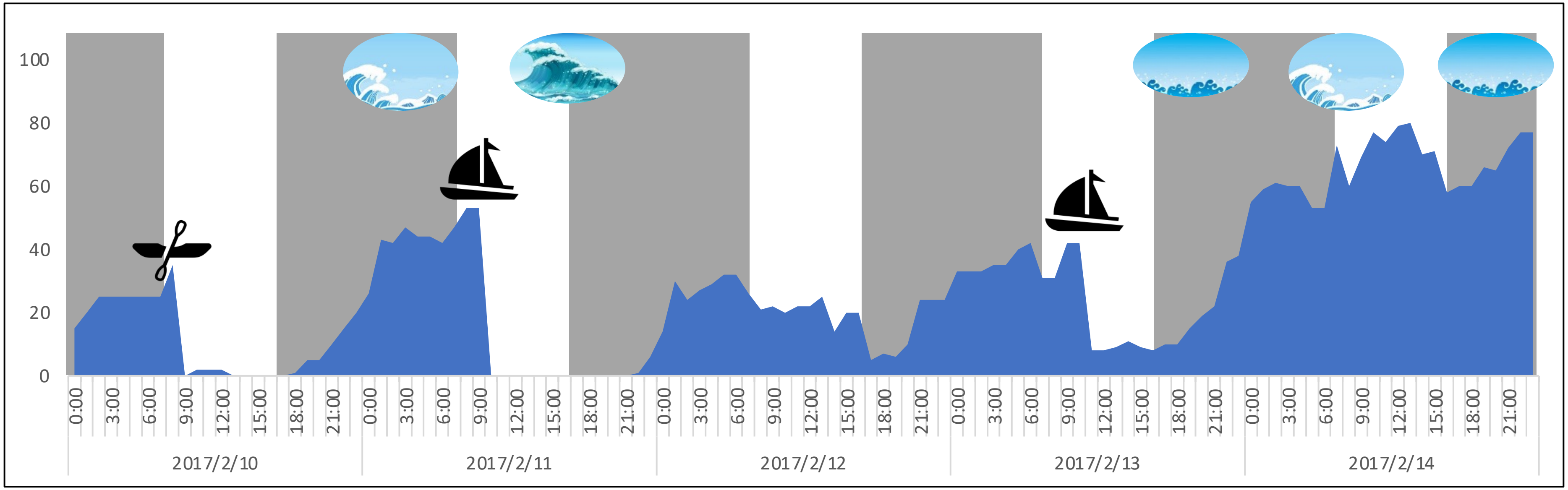

100
80
60
40
20
0
0:00
3:00
6:00
9:00
12:00
15:00
18:00
21:00
2017/2/10
2017/2/11
2017/2/12
2017/2/13
2017/2/14

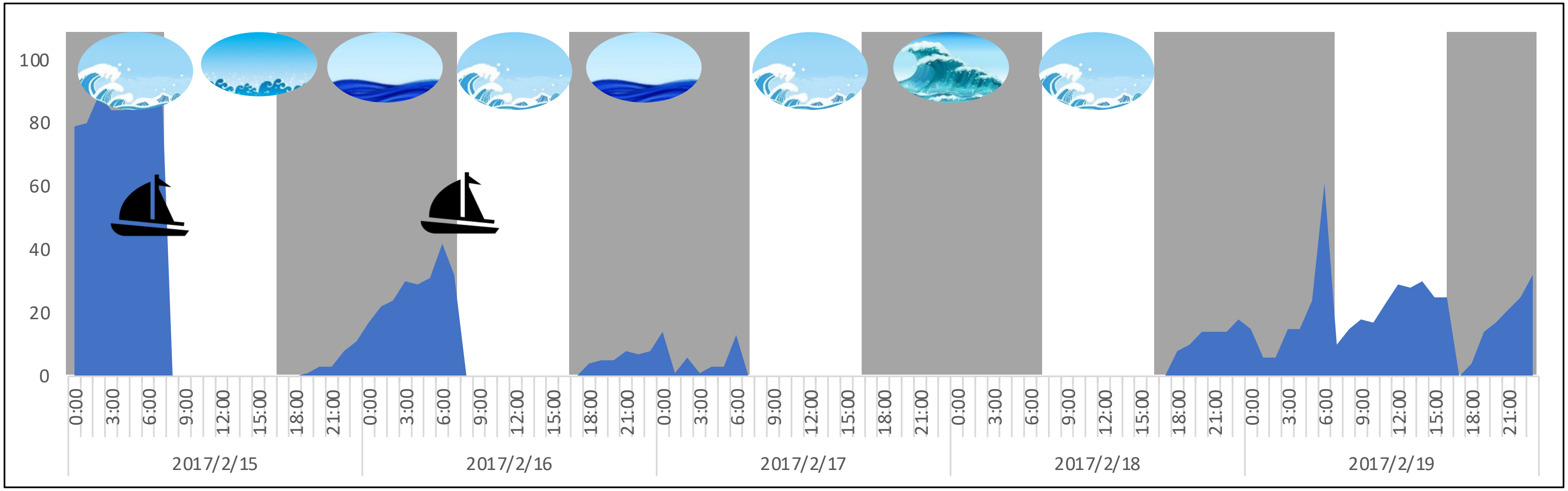

100
80
60
40
20
0
0:00
3:00
6:00
9:00
12:00
15:00
18:00
21:00
2017/2/15
2017/2/16
2017/2/17
2017/2/18
2017/2/19

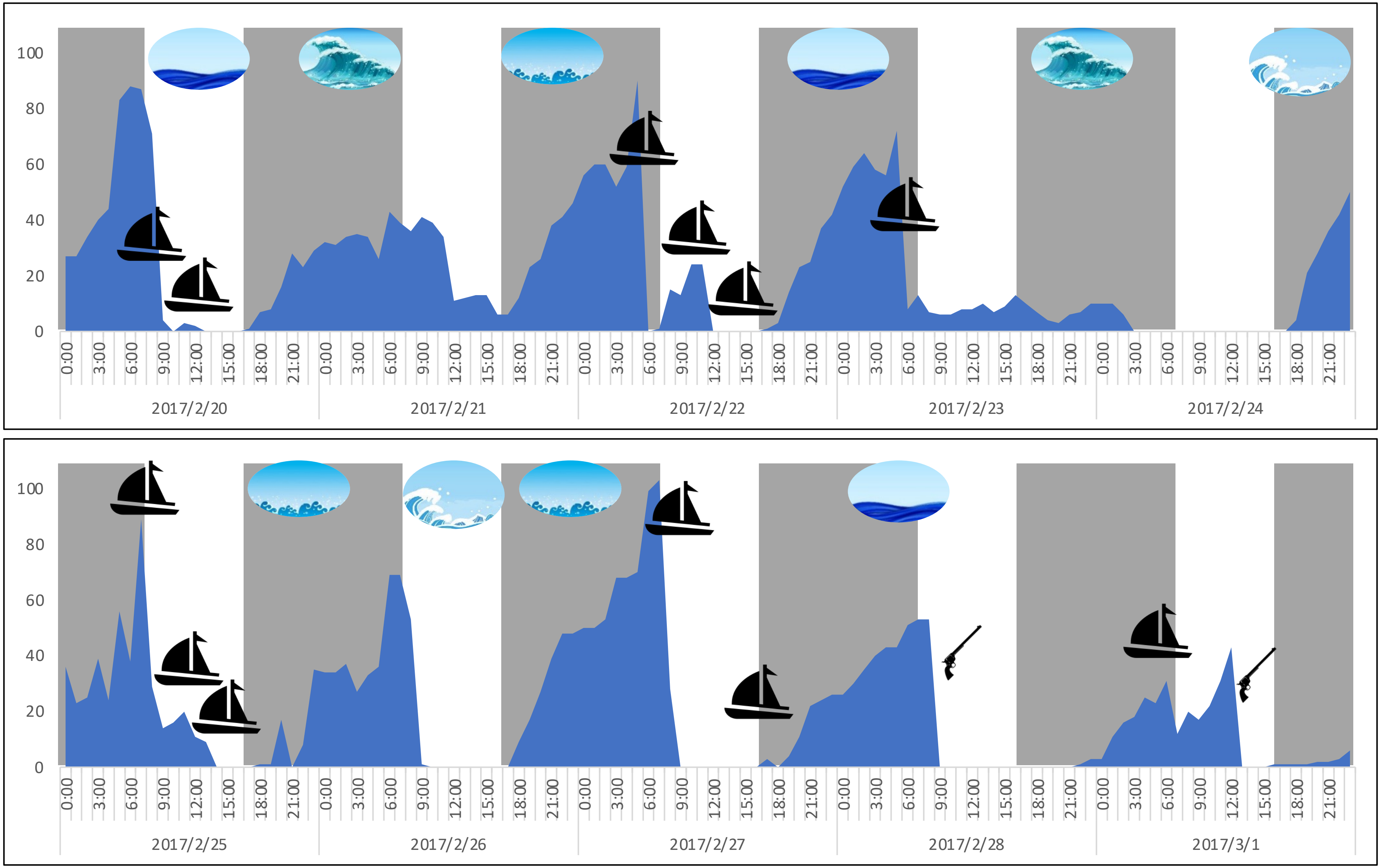
100
80
60
40
20
0
0:00
3:00
6:00
9:00
12:00
15:00
18:00
21:00
2017/2/20
2017/2/21
2017/2/22
2017/2/23
2017/2/24
2017/2/25
2017/2/26
2017/2/27
2017/2/28
2017/3/1

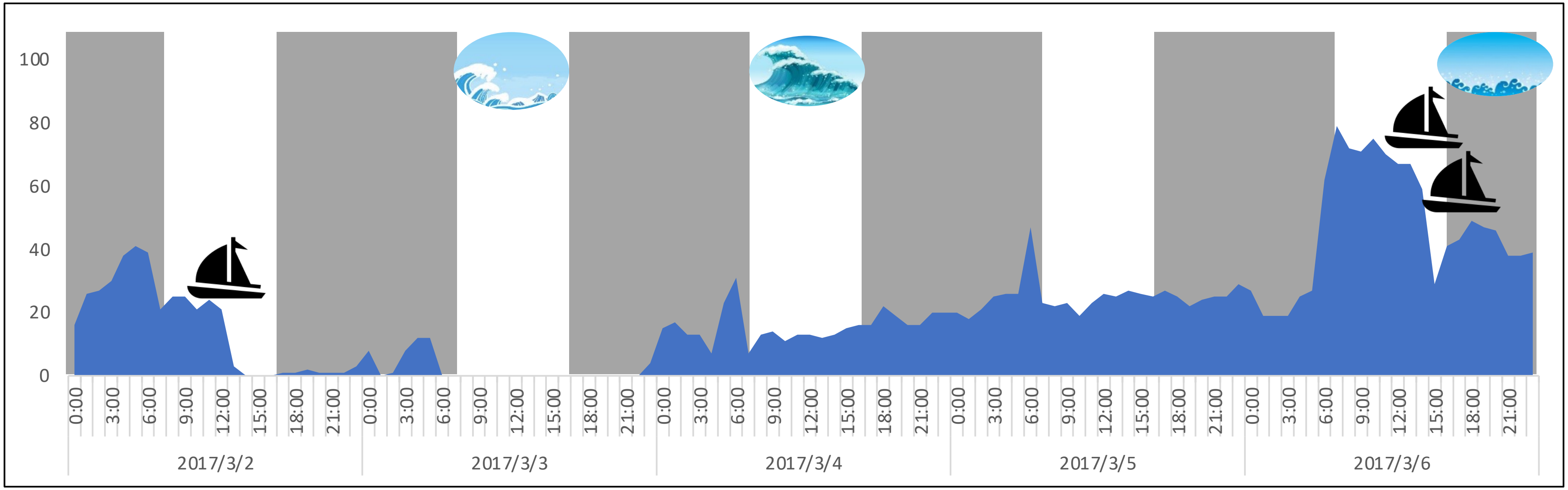
100
80
60
40
20
0
0:00
3:00
6:00
9:00
12:00
15:00
18:00
21:00
2017/3/2
2017/3/3
2017/3/4
2017/3/5
2017/3/6

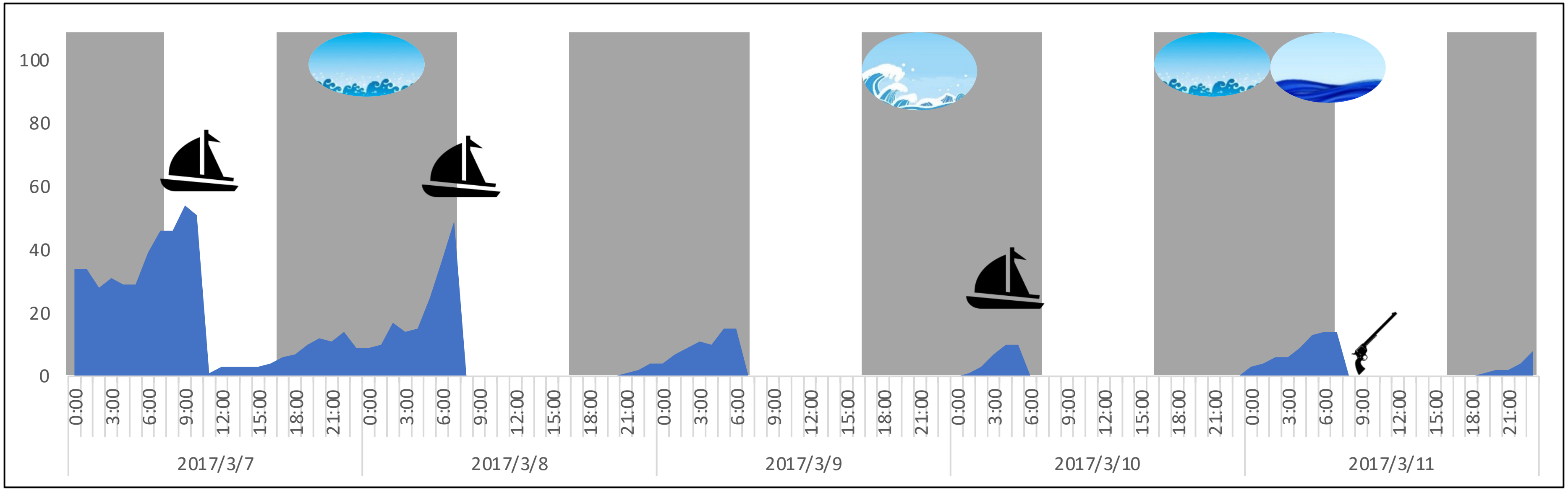
100
80
60
40
20
0
0:00
3:00
6:00
9:00
12:00
15:00
18:00
21:00
2017/3/7
2017/3/8
2017/3/9
2017/3/10
2017/3/11

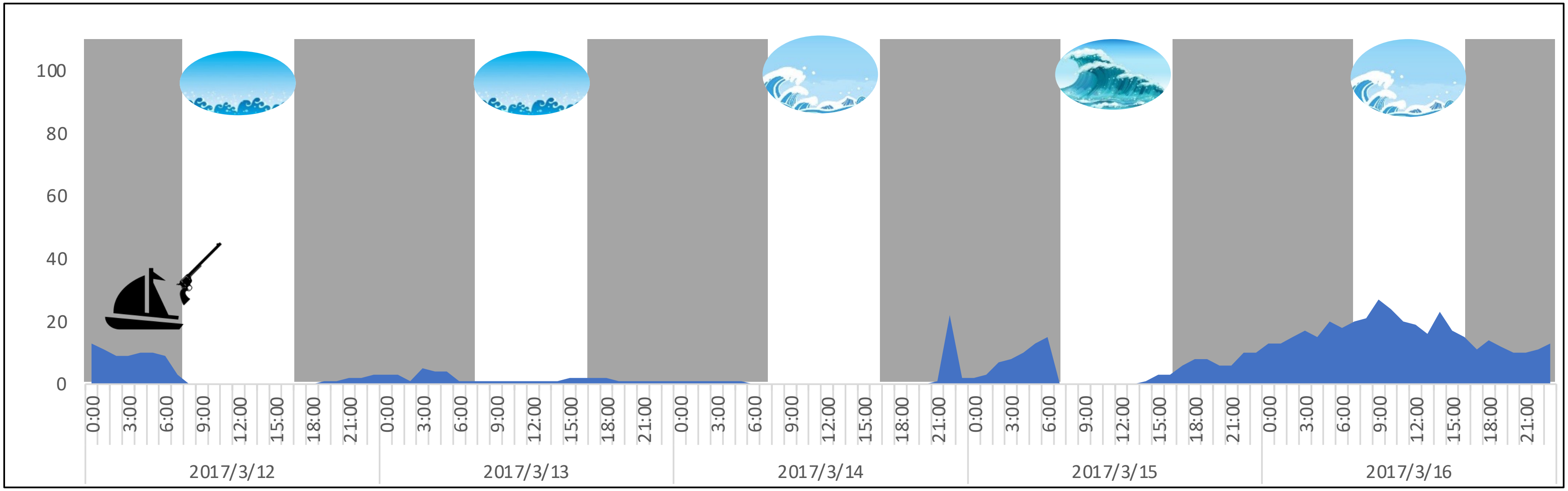
100
80
60
40
20
0
0:00 3:00 6:00 9:00 12:00 15:00 18:00 21:00
2017/3/12
2017/3/13
2017/3/14
2017/3/15
2017/3/16

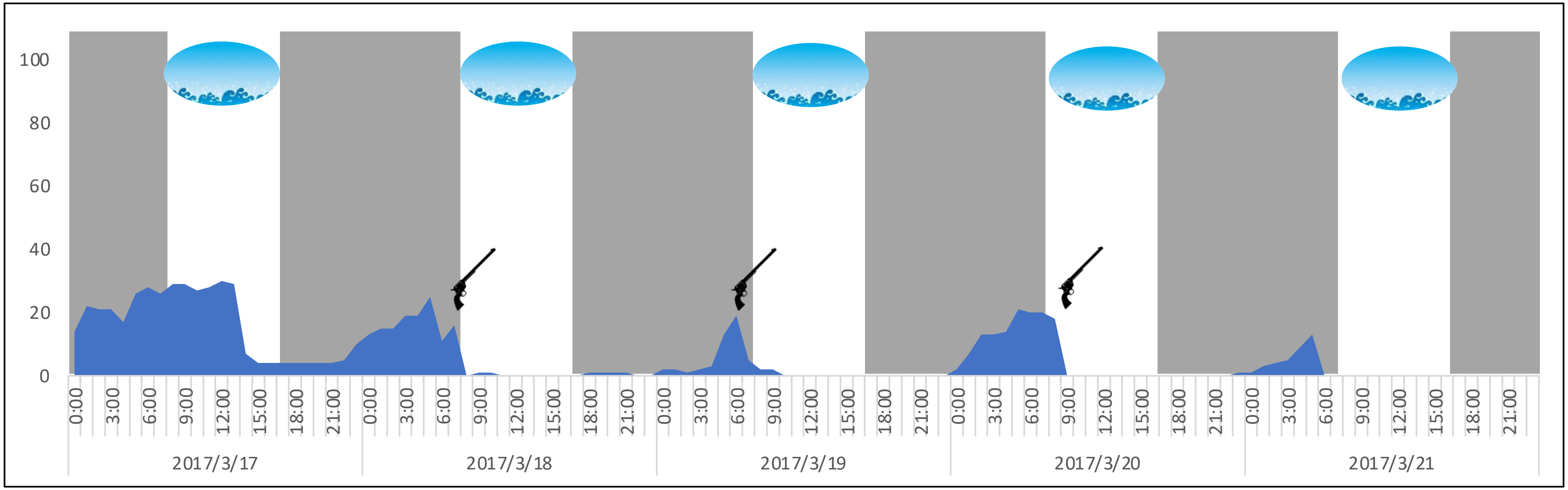
100
80
60
40
20
0
0:00 3:00 6:00 9:00 12:00 15:00 18:00 21:00
2017/3/17
2017/3/18
2017/3/19
2017/3/20
2017/3/21

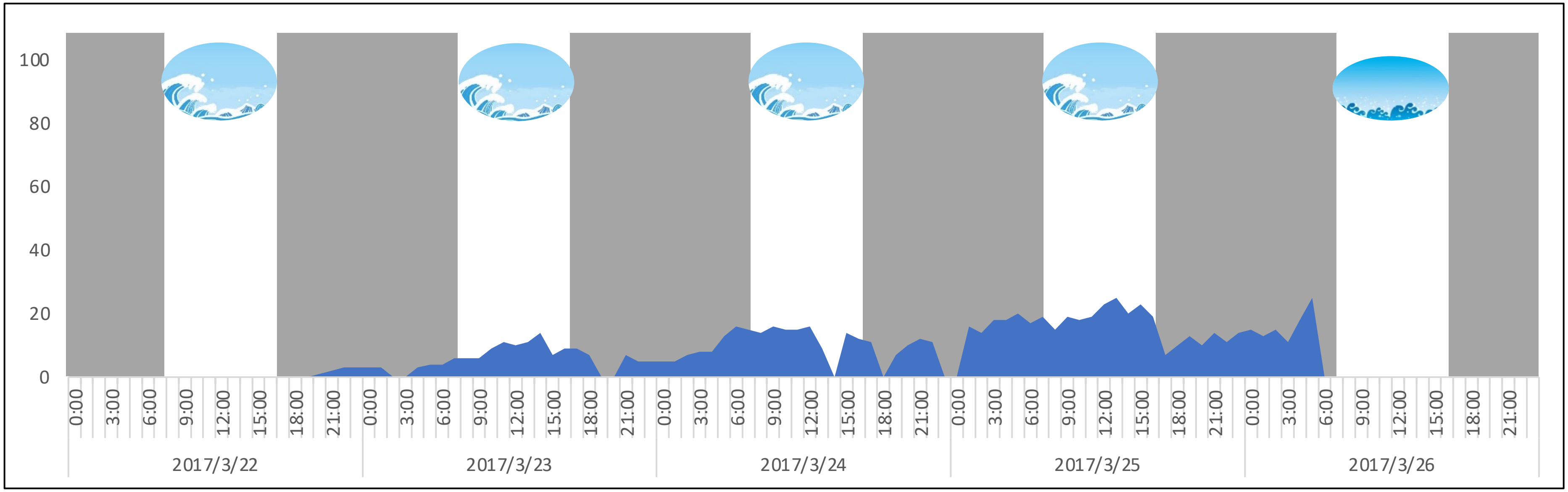

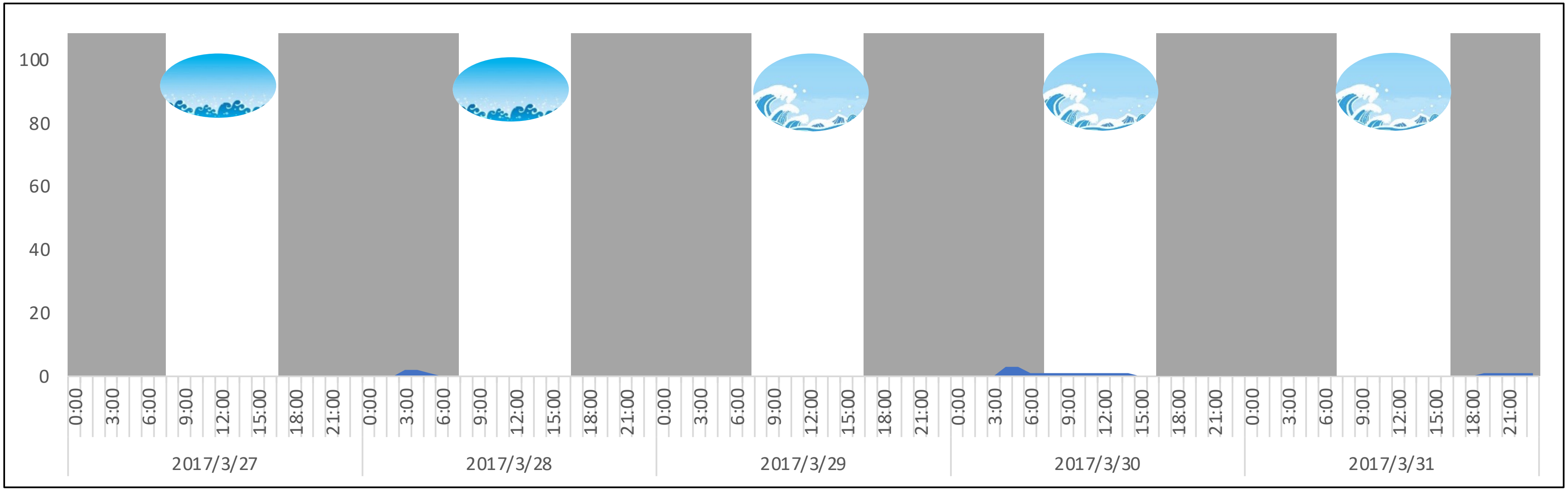

Figure S1. The hourly changes in the number of landed SSLs, weather conditions, and anthropogenic impacts from January 1 to March 31, 2017.
