# Supplementary material for: Human Impact on the Twenty-Four-Hour Patterns of Steller Sea Lions’ Use of a Haulout in Hokkaido, Japan
Source: Animals (Basel). 2024 Apr 27;14(9):1312. doi: 10.3390/ani14091312 (PMC11083395; doi:10.3390/ani14091312)
Supplement: Supplementary file 1 [file animals-14-01312-s001.zip › Figure S2_201801-201803.pdf]

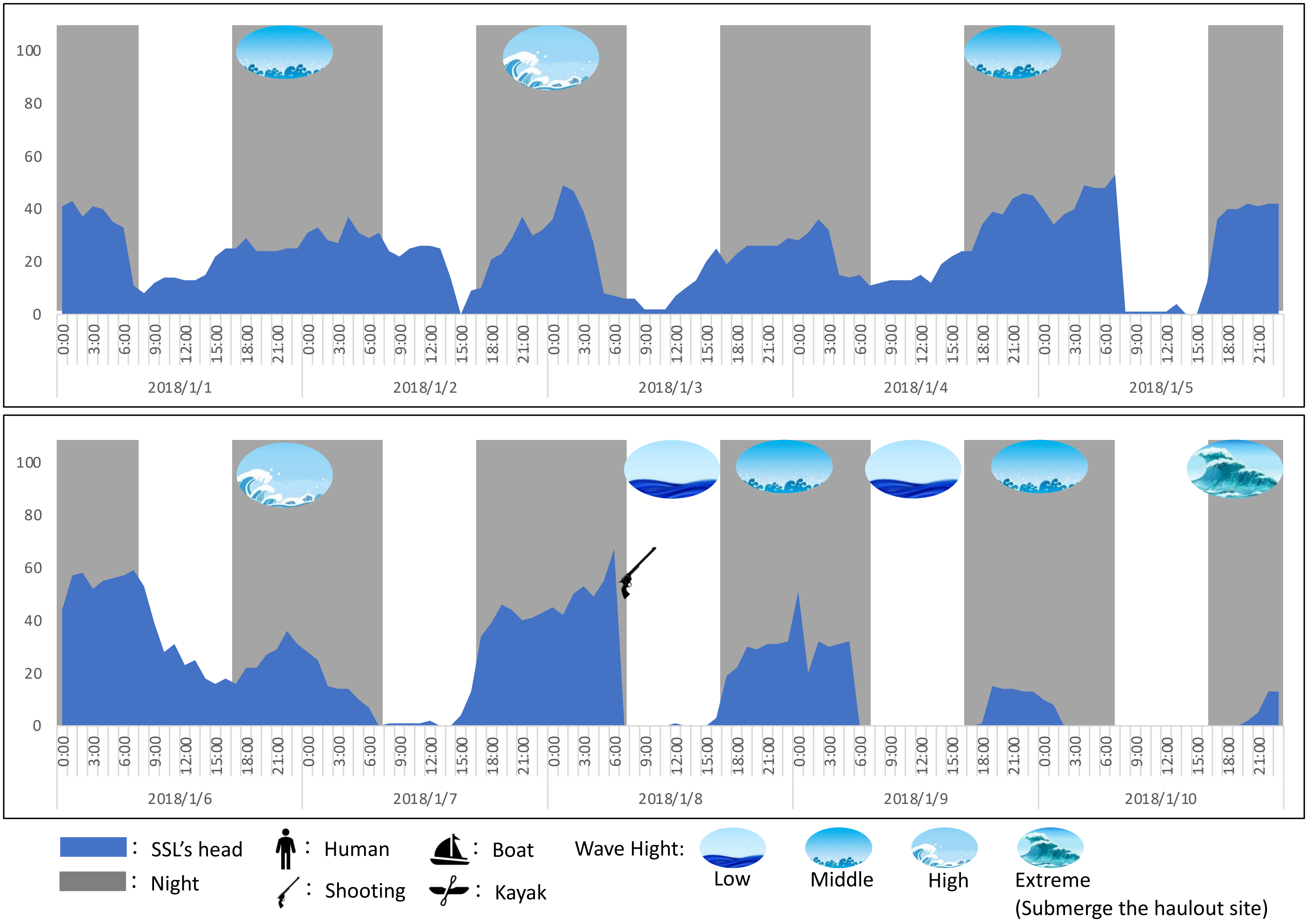

100
80
60
40
20
0
0:00 3:00 6:00 9:00 12:00 15:00 18:00 21:00
2018/1/1
2018/1/2
2018/1/3
2018/1/4
2018/1/5
2018/1/6
2018/1/7
2018/1/8
2018/1/9
2018/1/10
: SSL's head
: Night
: Human
: Shooting
: Boat
: Kayak
Wave Hight:
Low
Middle
High
Extreme
(Submerge the haulout site)

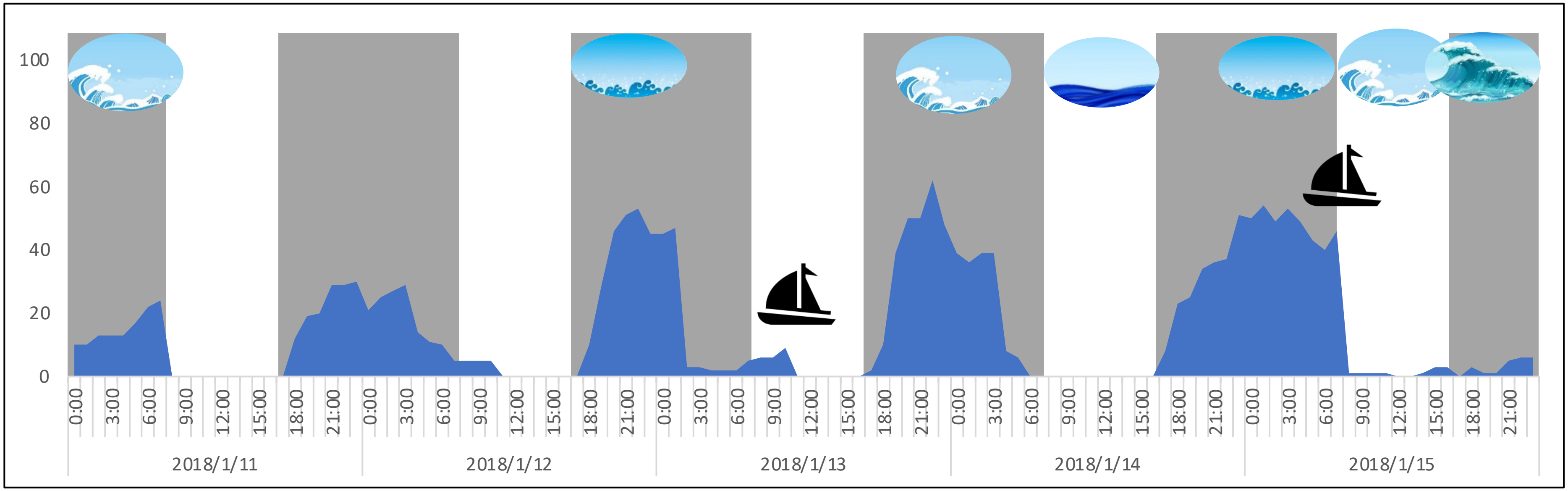

100
80
60
40
20
0
0:00
3:00
6:00
9:00
12:00
15:00
18:00
21:00
2018/1/11
2018/1/12
2018/1/13
2018/1/14
2018/1/15

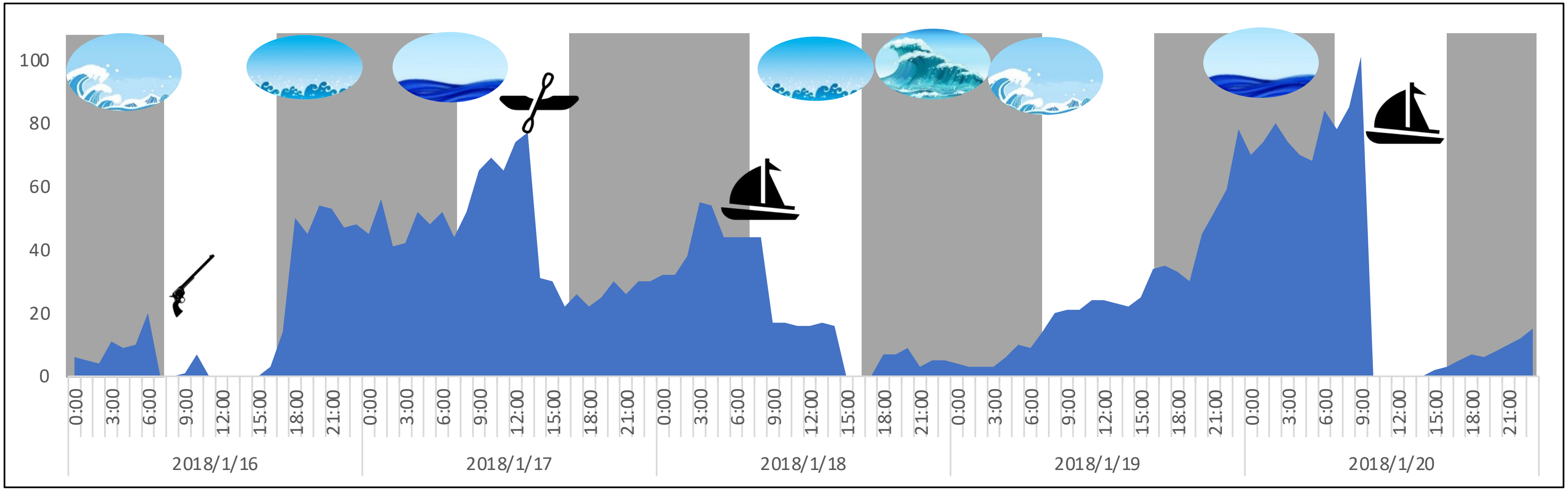

100
80
60
40
20
0
0:00
3:00
6:00
9:00
12:00
15:00
18:00
21:00
2018/1/16
2018/1/17
2018/1/18
2018/1/19
2018/1/20

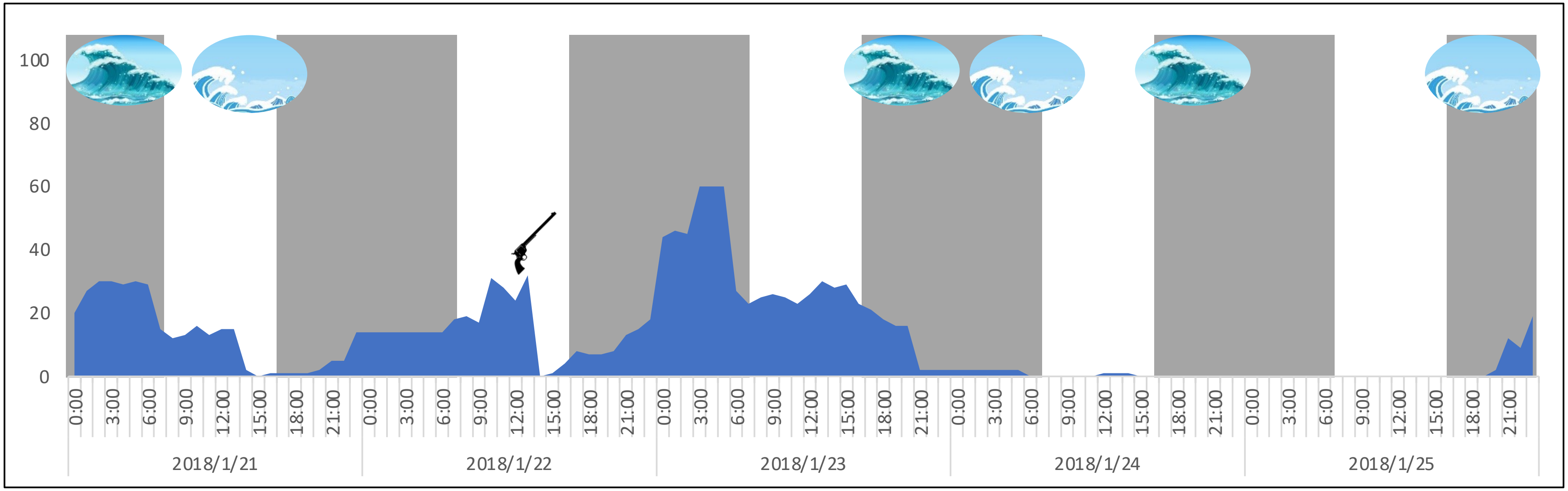

100
80
60
40
20
0
0:00
3:00
6:00
9:00
12:00
15:00
18:00
21:00
2018/1/21
2018/1/22
2018/1/23
2018/1/24
2018/1/25

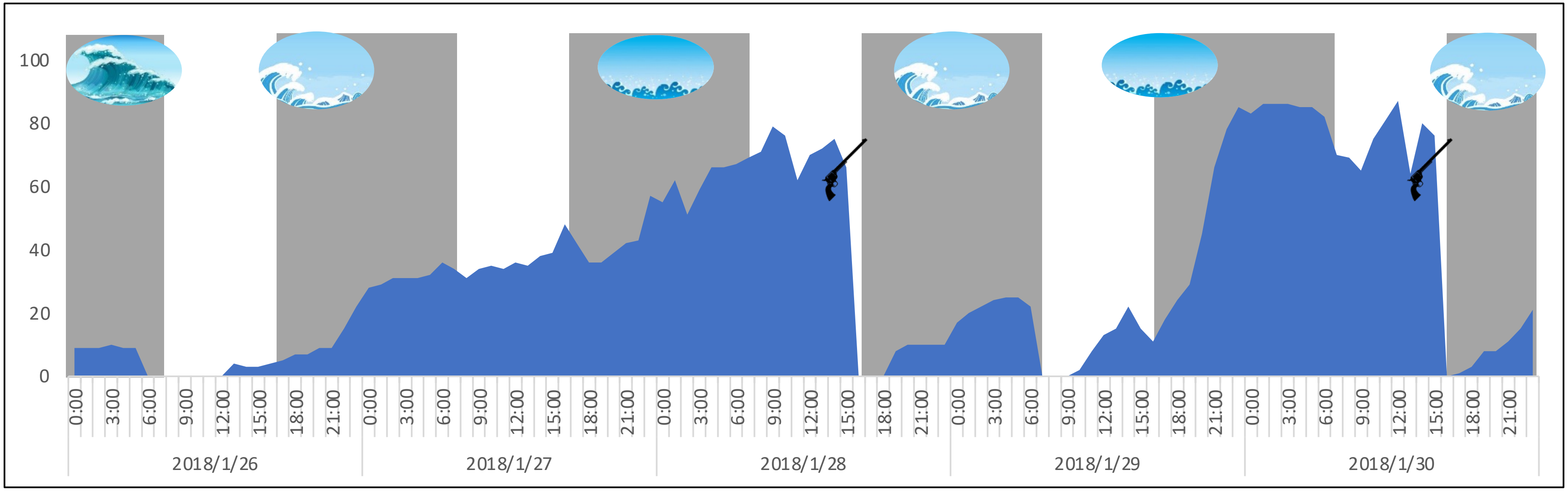

100
80
60
40
20
0
0:00
3:00
6:00
9:00
12:00
15:00
18:00
21:00
2018/1/26
2018/1/27
2018/1/28
2018/1/29
2018/1/30

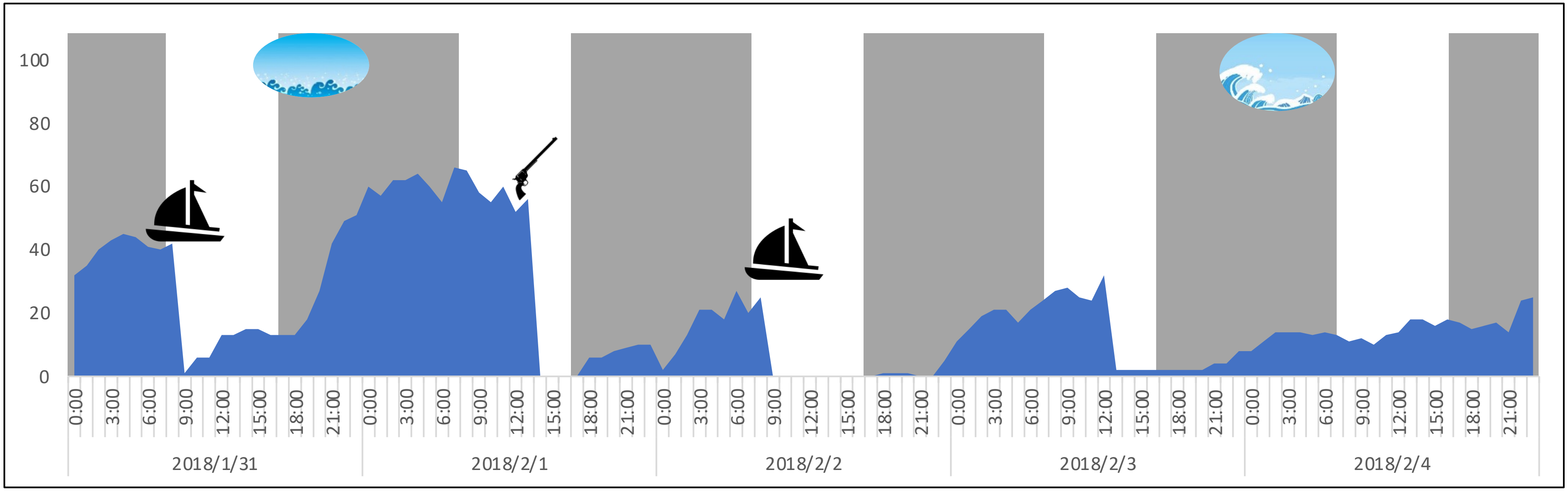
100
80
60
40
20
0
0:00 3:00 6:00 9:00 12:00 15:00 18:00 21:00
2018/1/31
2018/2/1
2018/2/2
2018/2/3
2018/2/4

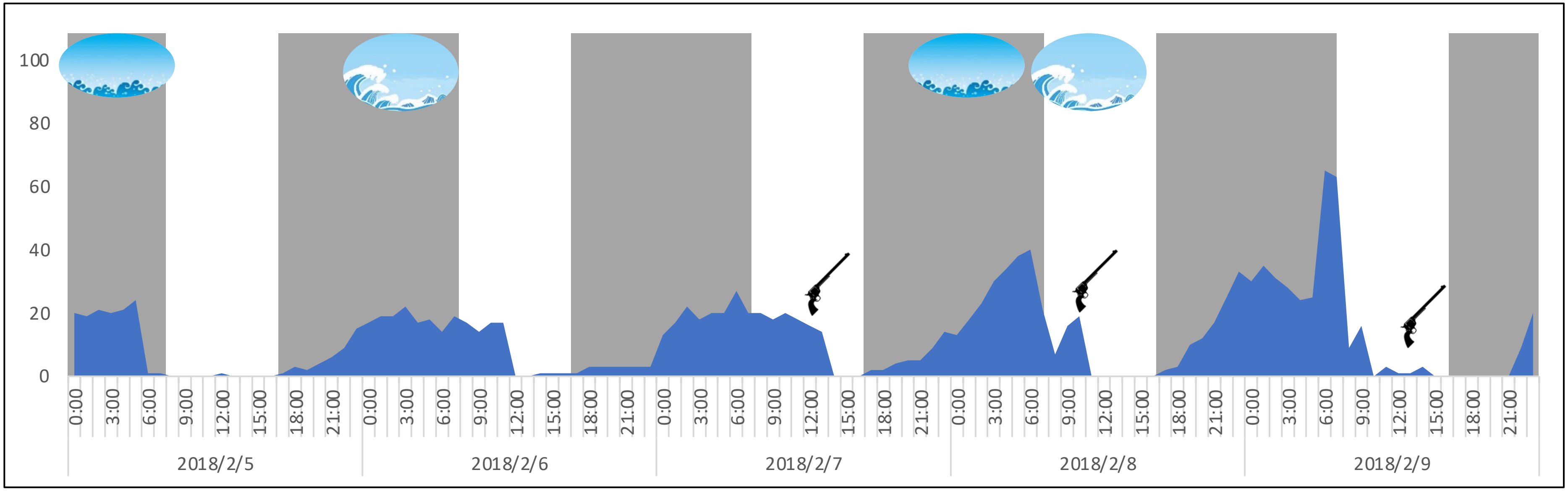
100
80
60
40
20
0
0:00 3:00 6:00 9:00 12:00 15:00 18:00 21:00
2018/2/5
2018/2/6
2018/2/7
2018/2/8
2018/2/9

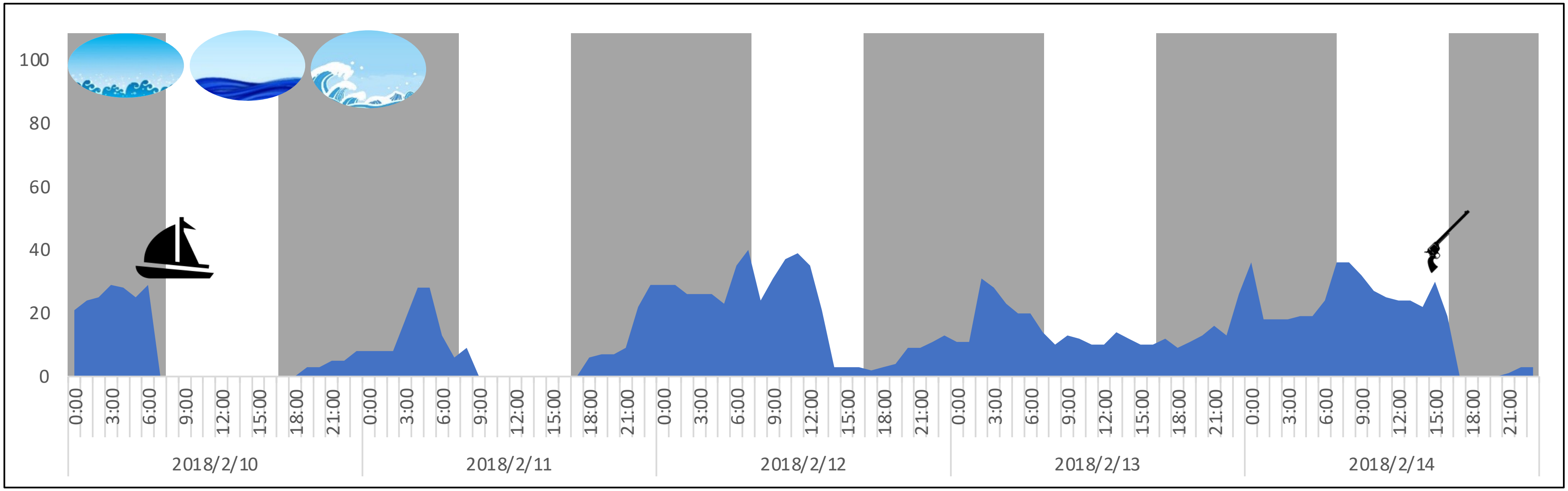
100
80
60
40
20
0
0:00 3:00 6:00 9:00 12:00 15:00 18:00 21:00
2018/2/10
2018/2/11
2018/2/12
2018/2/13
2018/2/14

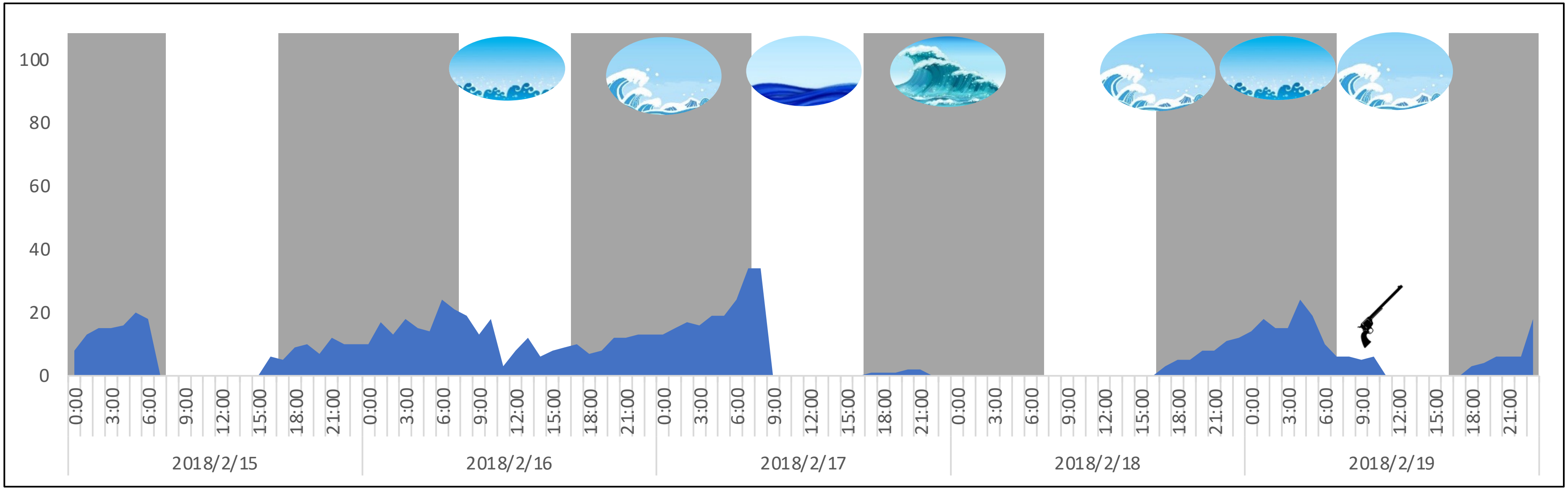
100
80
60
40
20
0
0:00 3:00 6:00 9:00 12:00 15:00 18:00 21:00
2018/2/15
2018/2/16
2018/2/17
2018/2/18
2018/2/19

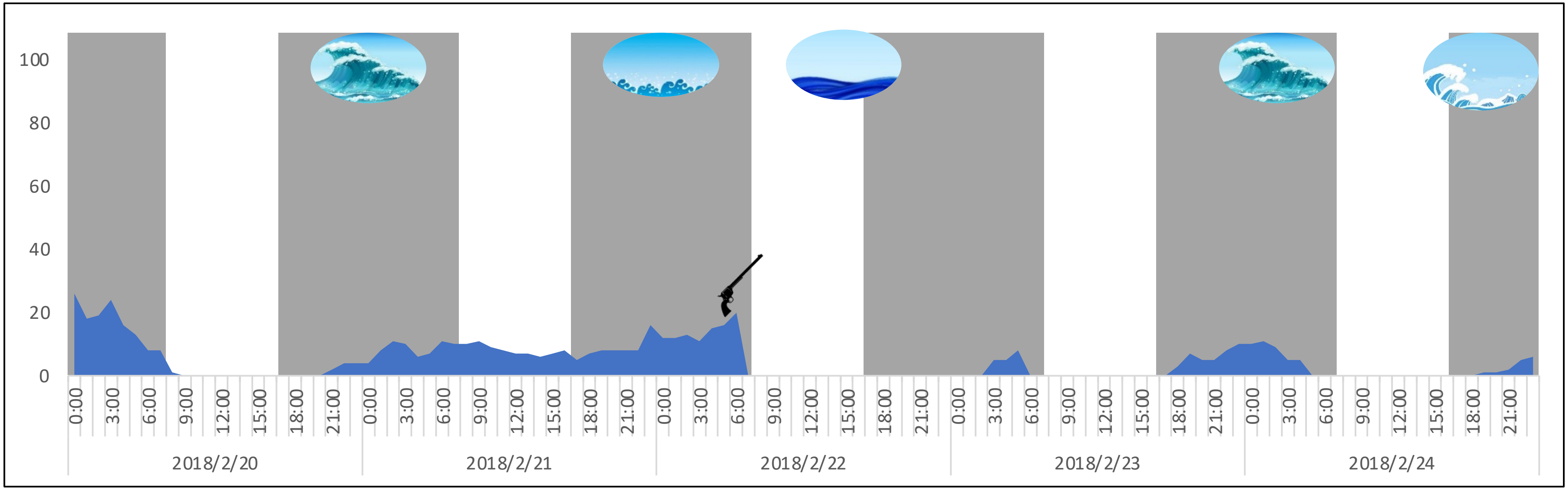

100
80
60
40
20
0
0:00 3:00 6:00 9:00 12:00 15:00 18:00 21:00
2018/2/20
2018/2/21
2018/2/22
2018/2/23
2018/2/24

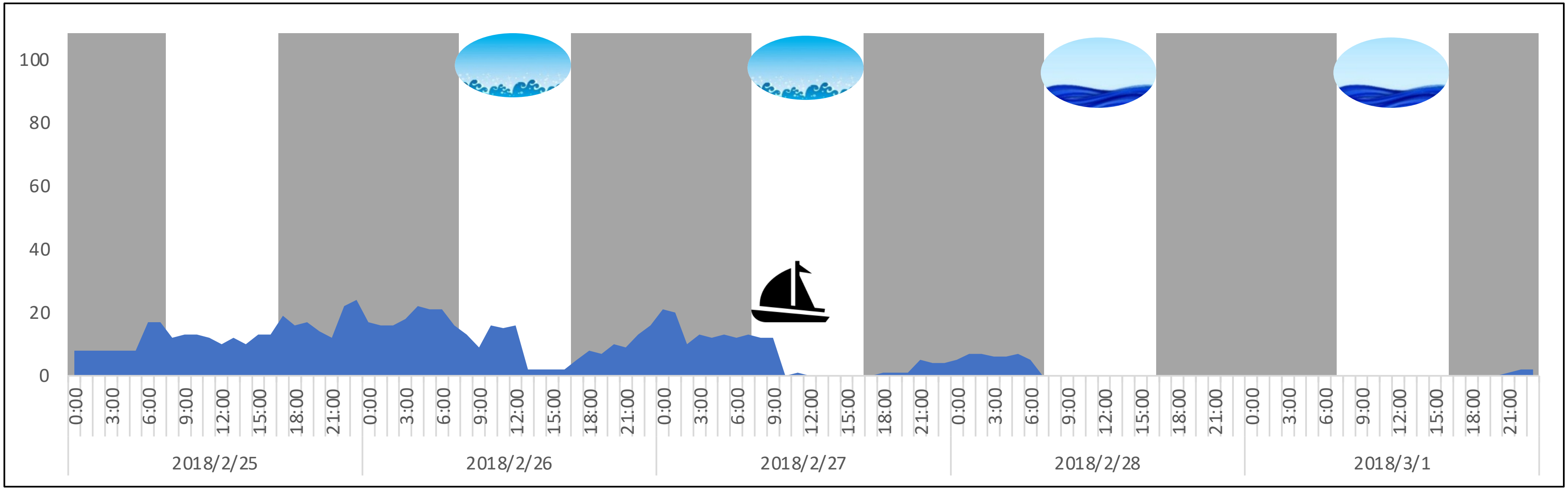

100
80
60
40
20
0
0:00 3:00 6:00 9:00 12:00 15:00 18:00 21:00
2018/2/25
2018/2/26
2018/2/27
2018/2/28
2018/3/1

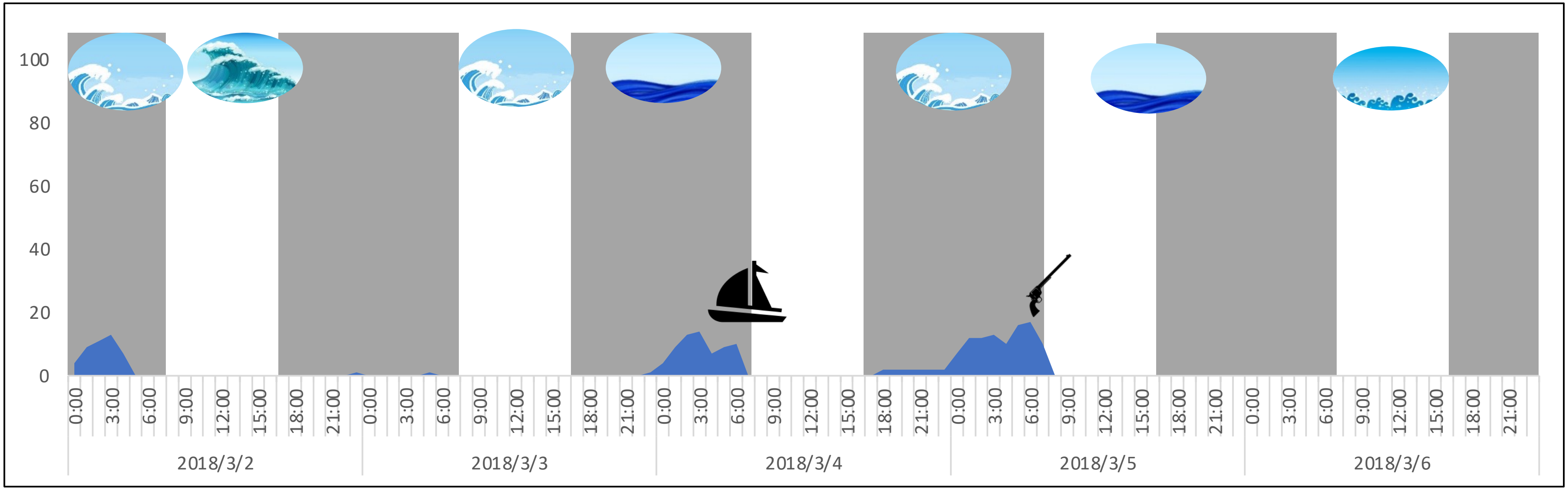
100
80
60
40
20
0
0:00 3:00 6:00 9:00 12:00 15:00 18:00 21:00
2018/3/2
2018/3/3
2018/3/4
2018/3/5
2018/3/6

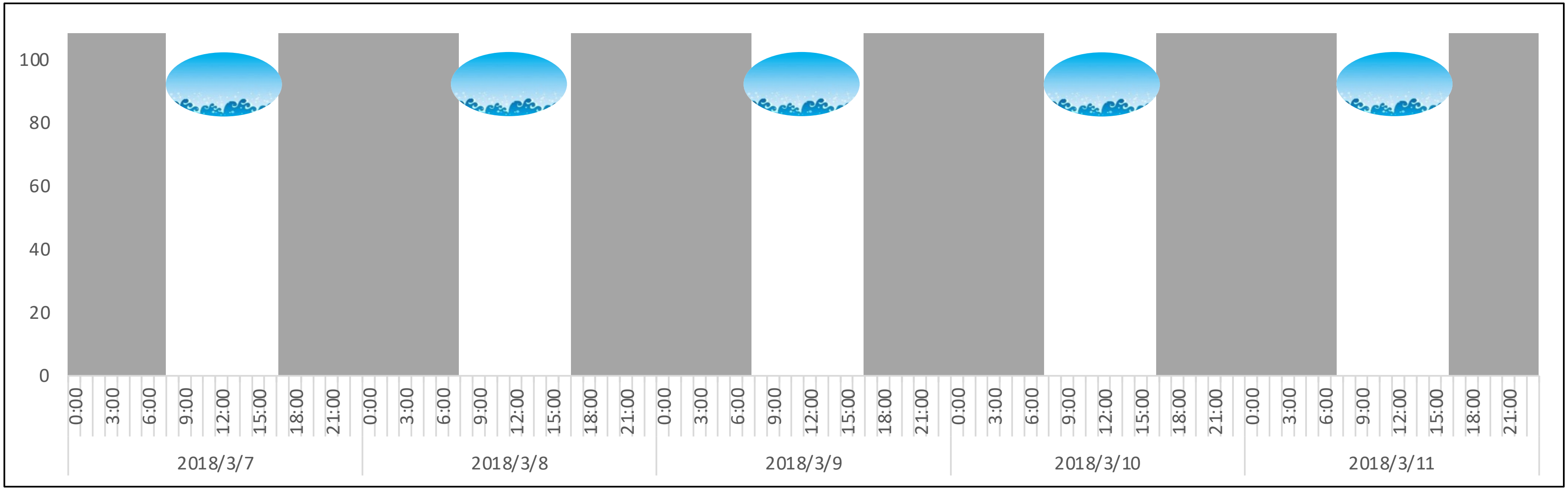
100
80
60
40
20
0
0:00 3:00 6:00 9:00 12:00 15:00 18:00 21:00
2018/3/7
2018/3/8
2018/3/9
2018/3/10
2018/3/11

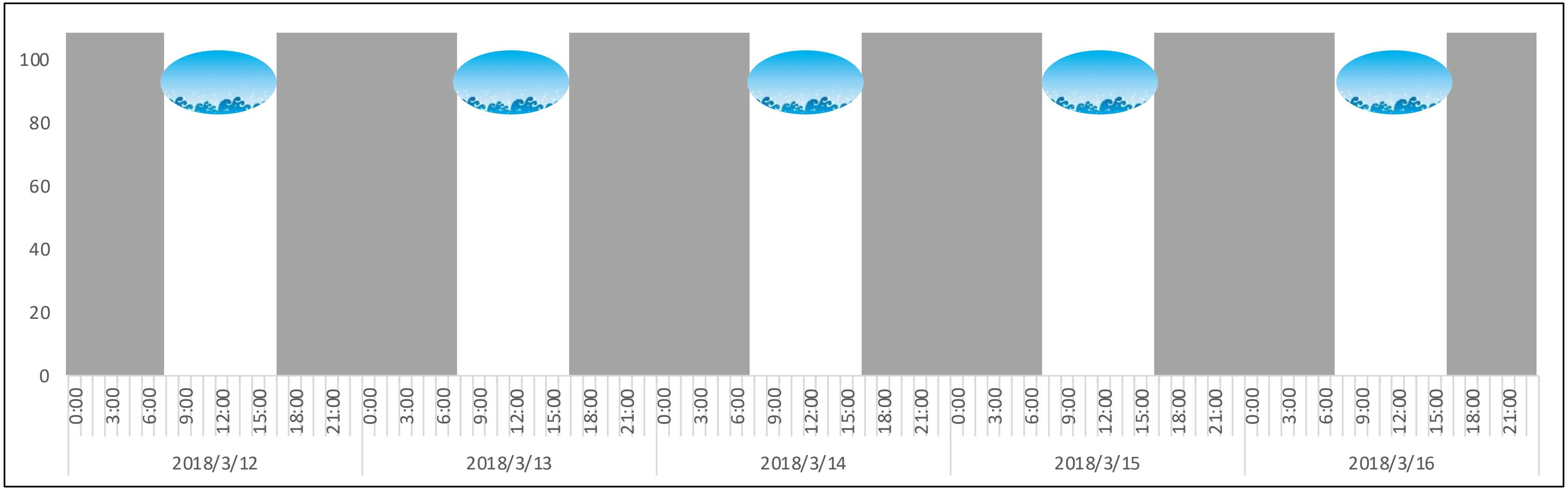

100
80
60
40
20
0
0:00
3:00
6:00
9:00
12:00
15:00
18:00
21:00
2018/3/12
2018/3/13
2018/3/14
2018/3/15
2018/3/16

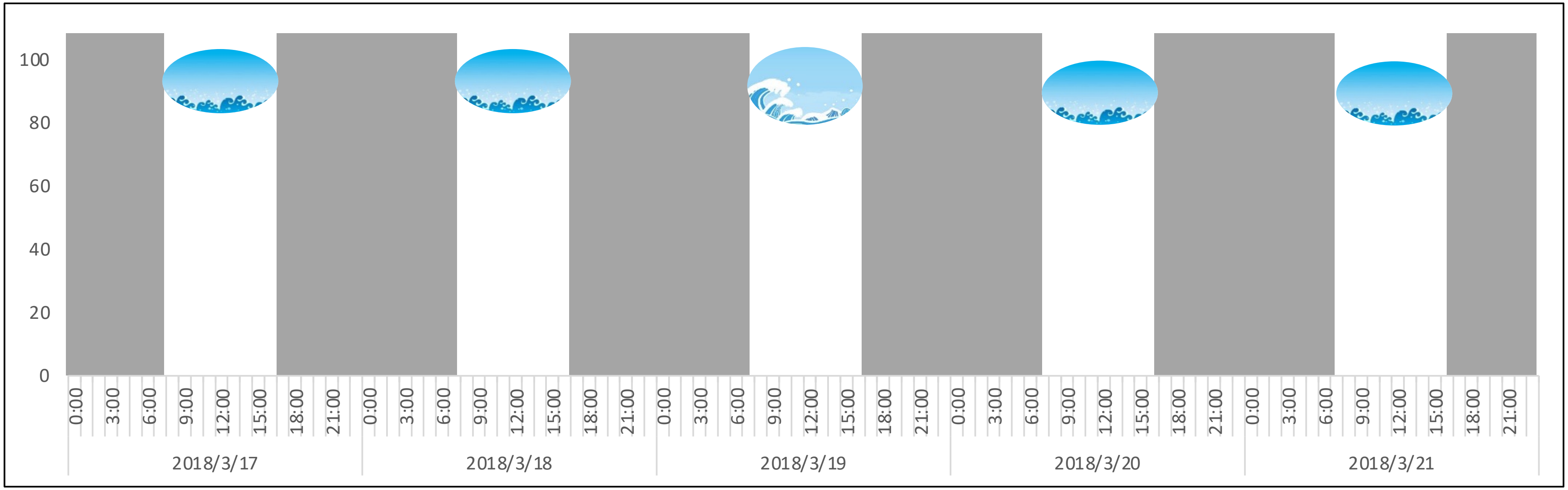

100
80
60
40
20
0
0:00
3:00
6:00
9:00
12:00
15:00
18:00
21:00
2018/3/17
2018/3/18
2018/3/19
2018/3/20
2018/3/21

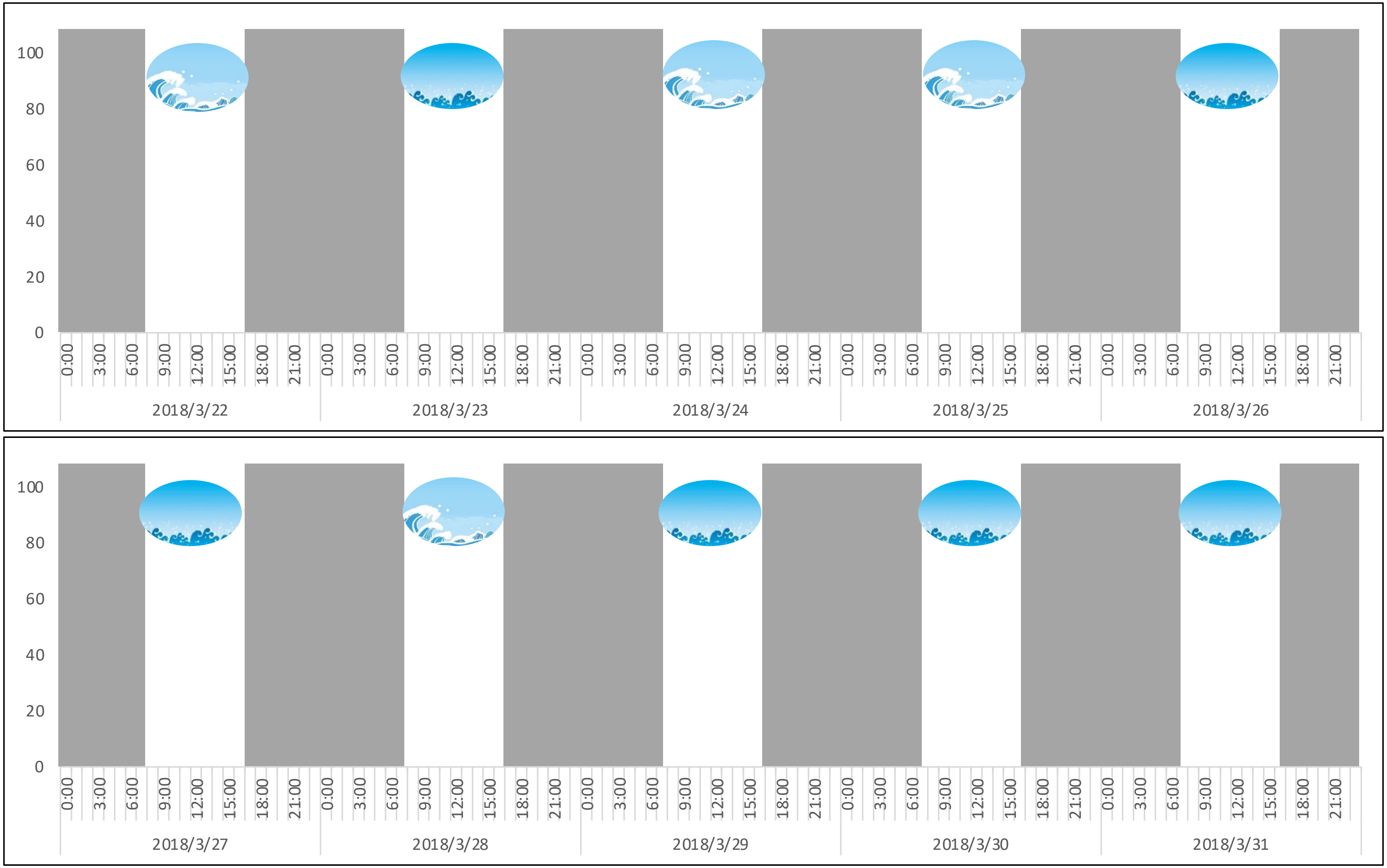

Figure S2. The hourly changes in the number of landed SSLs, weather conditions, and anthropogenic impacts from January 1 to March 31, 2018.
