# Supplementary material for: Human Impact on the Twenty-Four-Hour Patterns of Steller Sea Lions’ Use of a Haulout in Hokkaido, Japan
Source: Animals (Basel). 2024 Apr 27;14(9):1312. doi: 10.3390/ani14091312 (PMC11083395; doi:10.3390/ani14091312)
Supplement: Supplementary file 1 [file animals-14-01312-s001.zip › Figure S3_201901-201903.pdf]

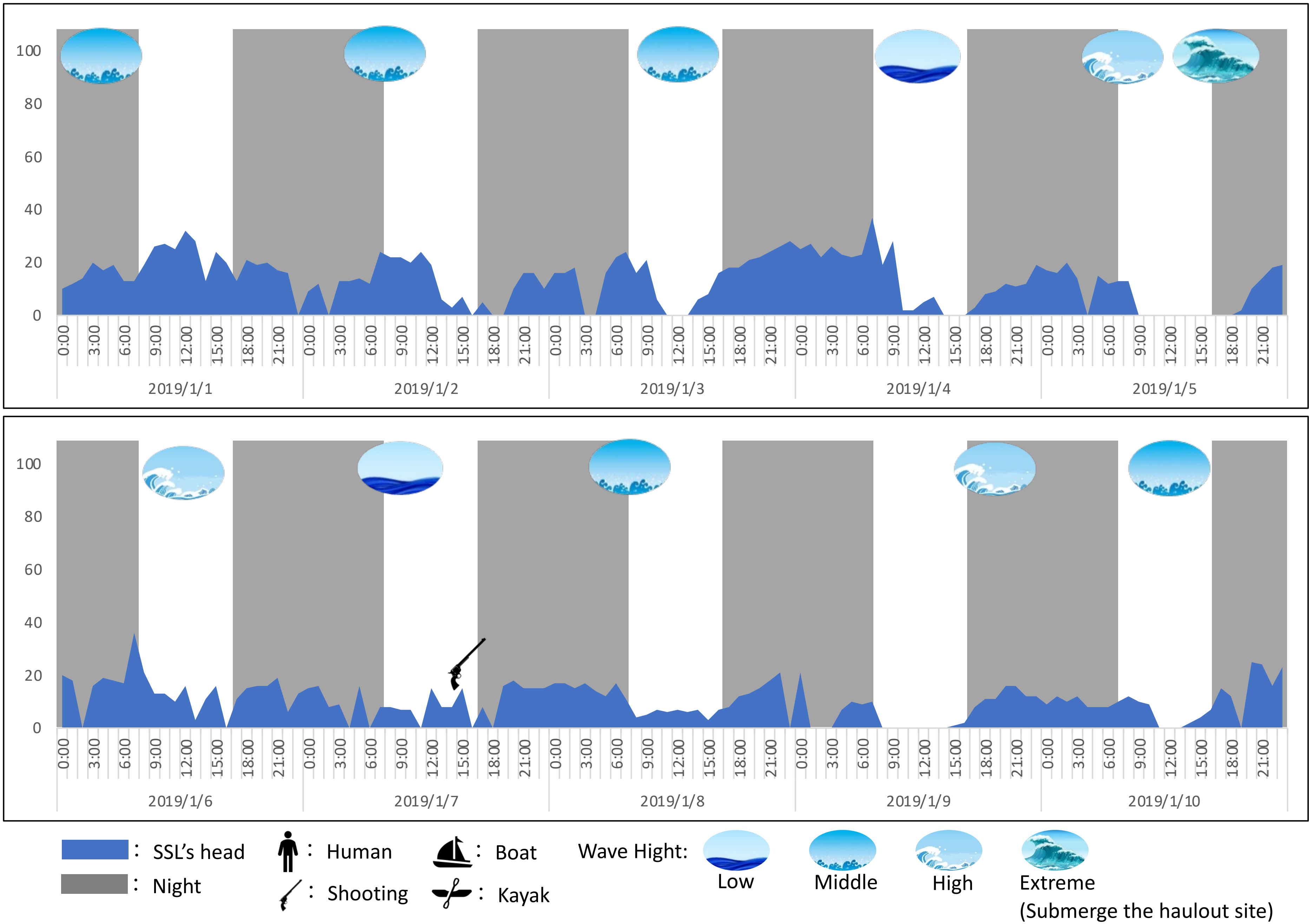
100
80
60
40
20
0
0:00 3:00 6:00 9:00 12:00 15:00 18:00 21:00
2019/1/1
2019/1/2
2019/1/3
2019/1/4
2019/1/5
2019/1/6
2019/1/7
2019/1/8
2019/1/9
2019/1/10
: SSL's head
: Night
: Human
: Shooting
: Boat
: Kayak
Wave Hight:
Low
Middle
High
Extreme
(Submerge the haulout site)

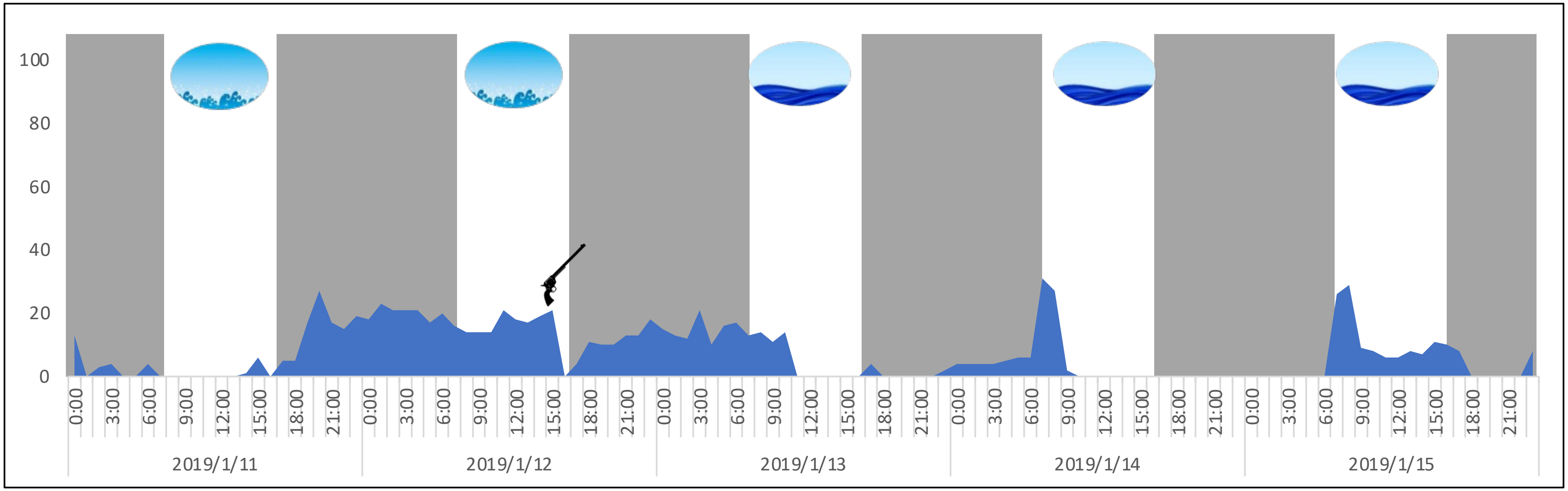

100
80
60
40
20
0
0:00
3:00
6:00
9:00
12:00
15:00
18:00
21:00
2019/1/11
2019/1/12
2019/1/13
2019/1/14
2019/1/15

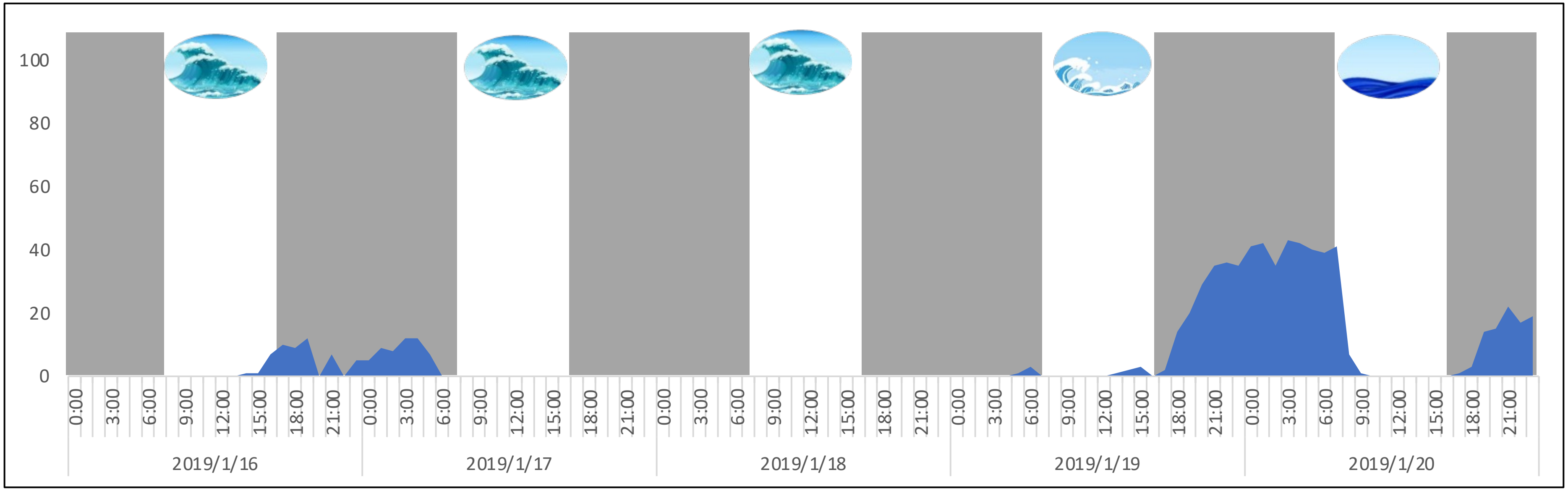

100
80
60
40
20
0
0:00
3:00
6:00
9:00
12:00
15:00
18:00
21:00
2019/1/16
2019/1/17
2019/1/18
2019/1/19
2019/1/20

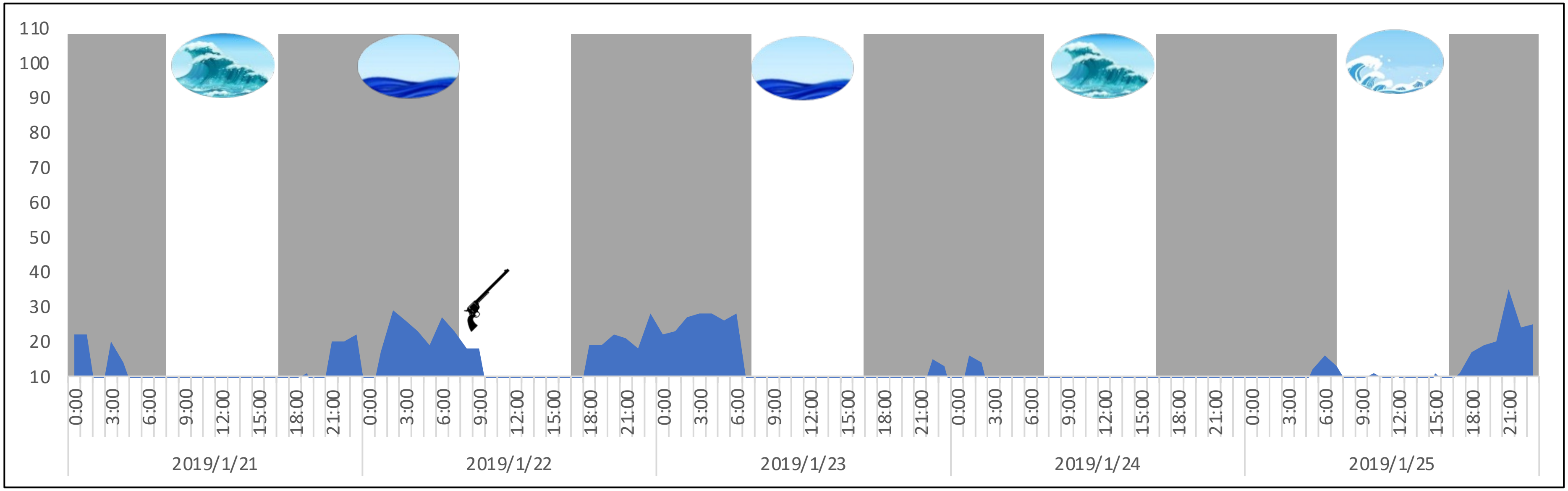
110
100
90
80
70
60
50
40
30
20
10
0:00 3:00 6:00 9:00 12:00 15:00 18:00 21:00
2019/1/21
2019/1/22
2019/1/23
2019/1/24
2019/1/25

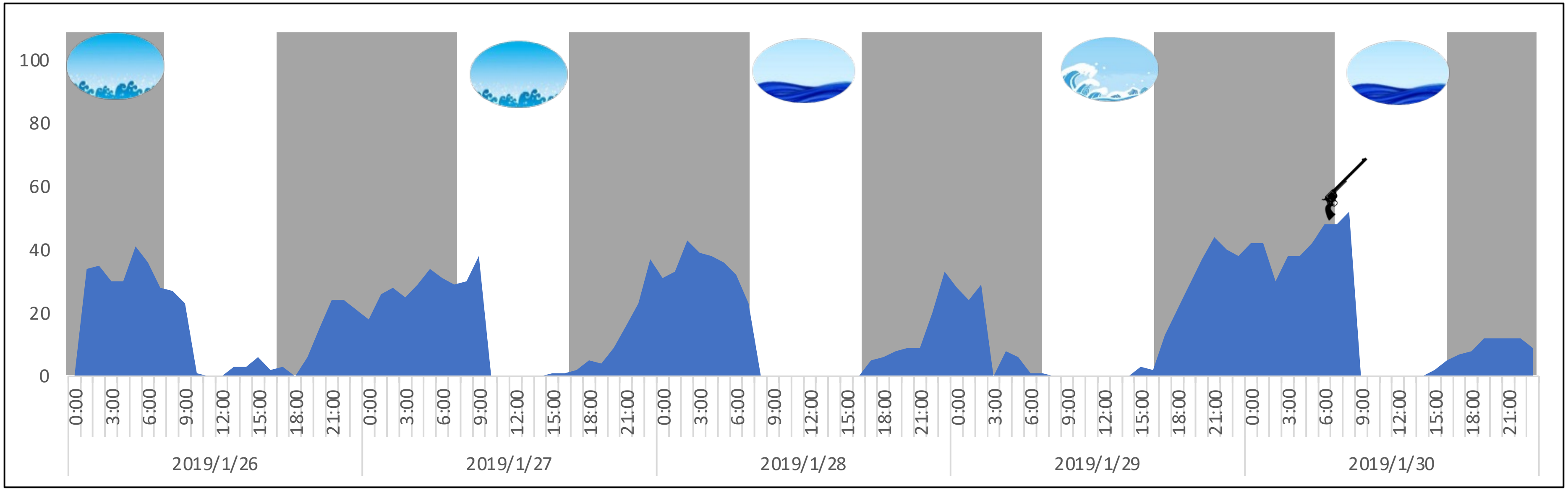
100
80
60
40
20
0
0:00 3:00 6:00 9:00 12:00 15:00 18:00 21:00
2019/1/26
2019/1/27
2019/1/28
2019/1/29
2019/1/30

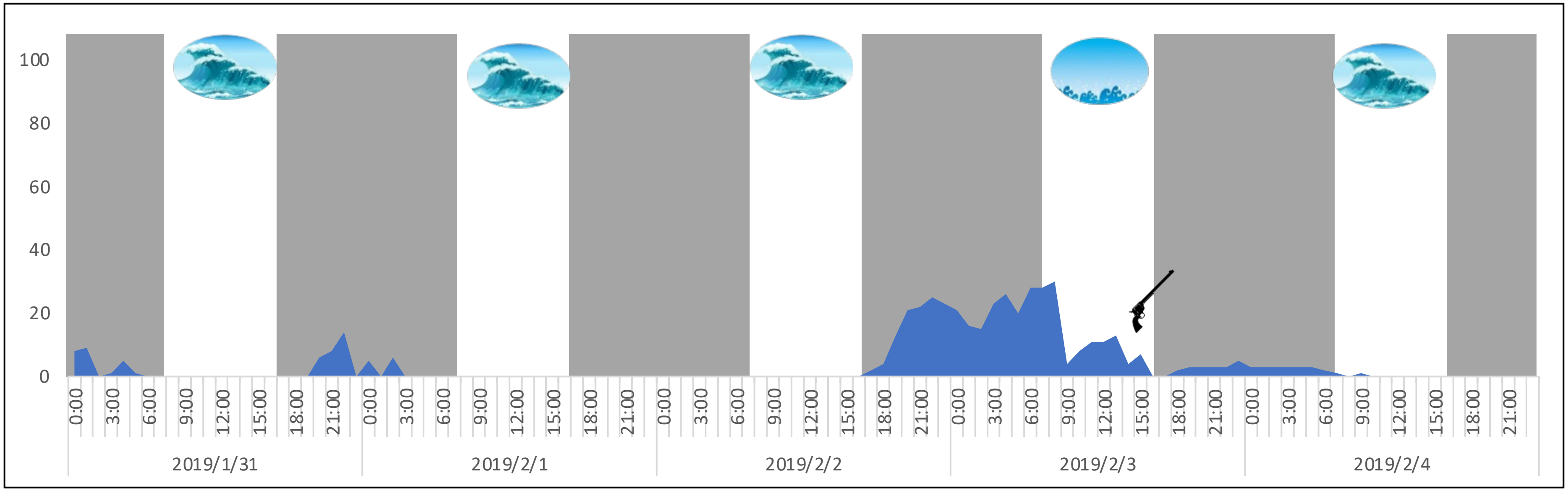

100
80
60
40
20
0
0:00
3:00
6:00
9:00
12:00
15:00
18:00
21:00
2019/1/31
2019/2/1
2019/2/2
2019/2/3
2019/2/4

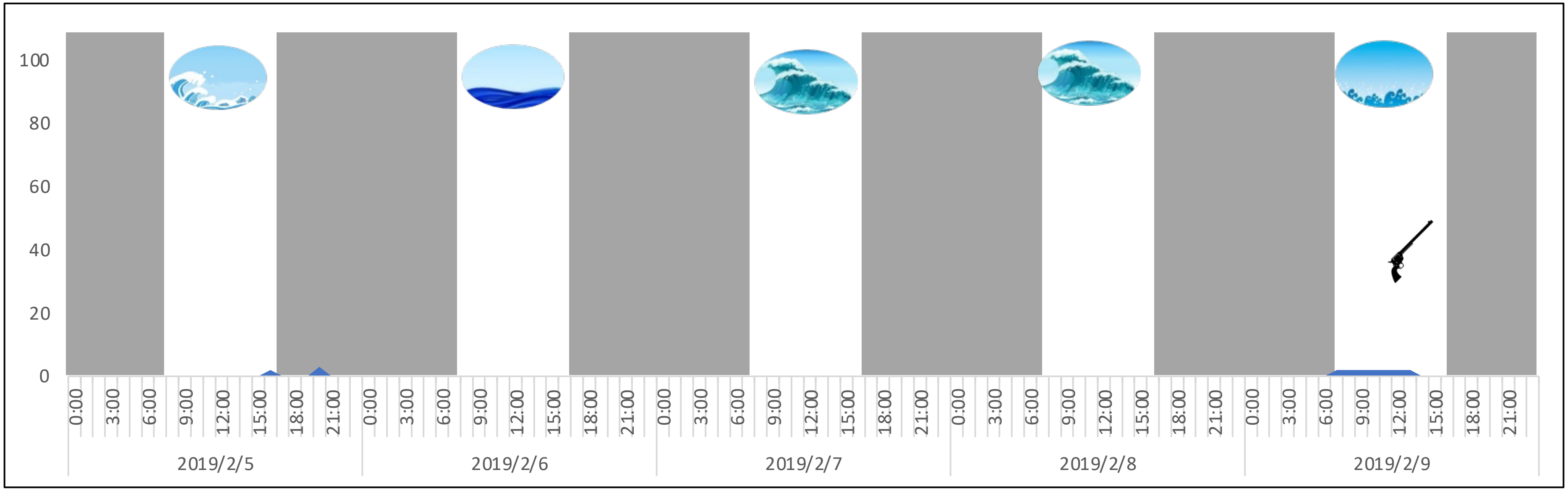

100
80
60
40
20
0
0:00
3:00
6:00
9:00
12:00
15:00
18:00
21:00
2019/2/5
2019/2/6
2019/2/7
2019/2/8
2019/2/9

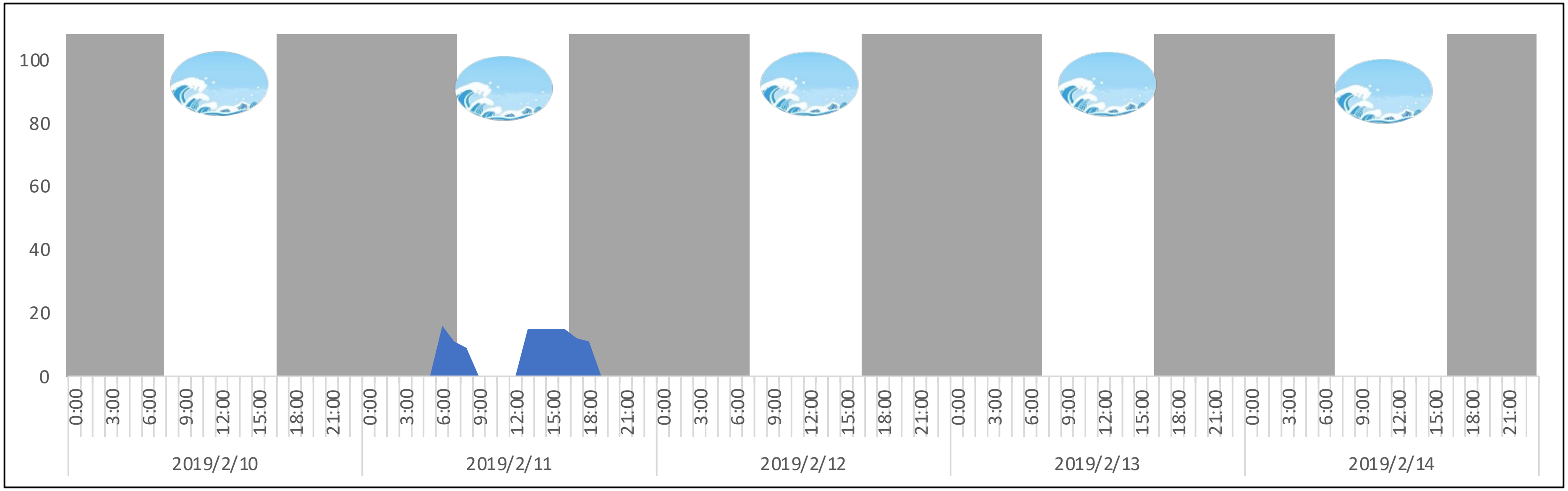
100
80
60
40
20
0
0:00 3:00 6:00 9:00 12:00 15:00 18:00 21:00
2019/2/10
2019/2/11
2019/2/12
2019/2/13
2019/2/14

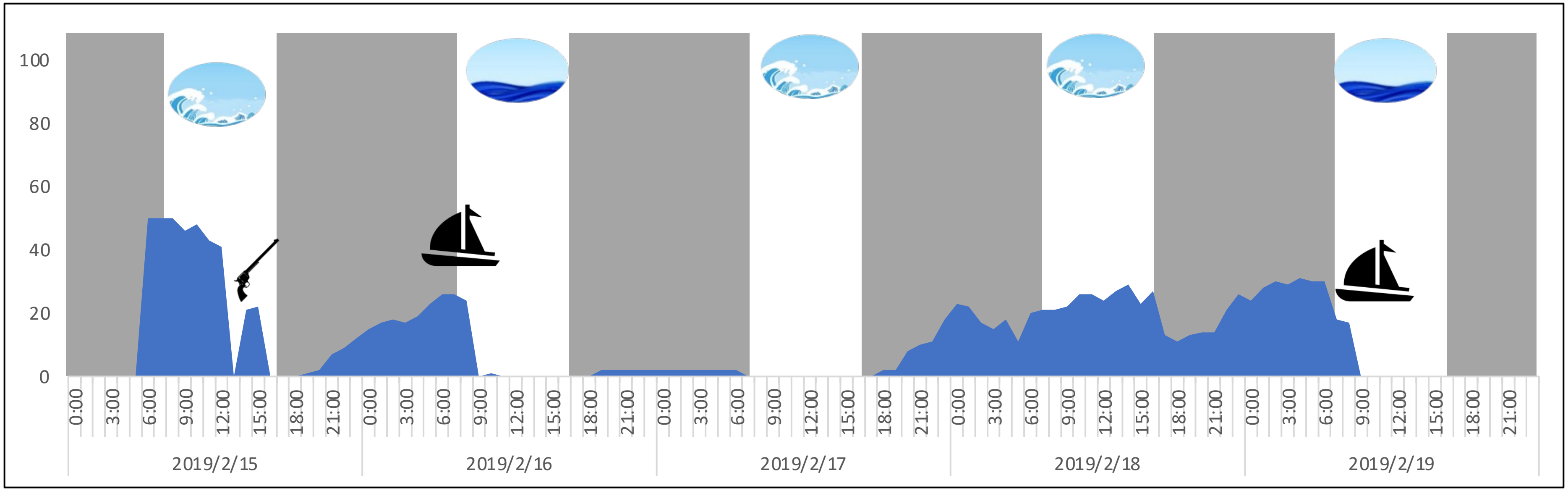
100
80
60
40
20
0
0:00 3:00 6:00 9:00 12:00 15:00 18:00 21:00
2019/2/15
2019/2/16
2019/2/17
2019/2/18
2019/2/19

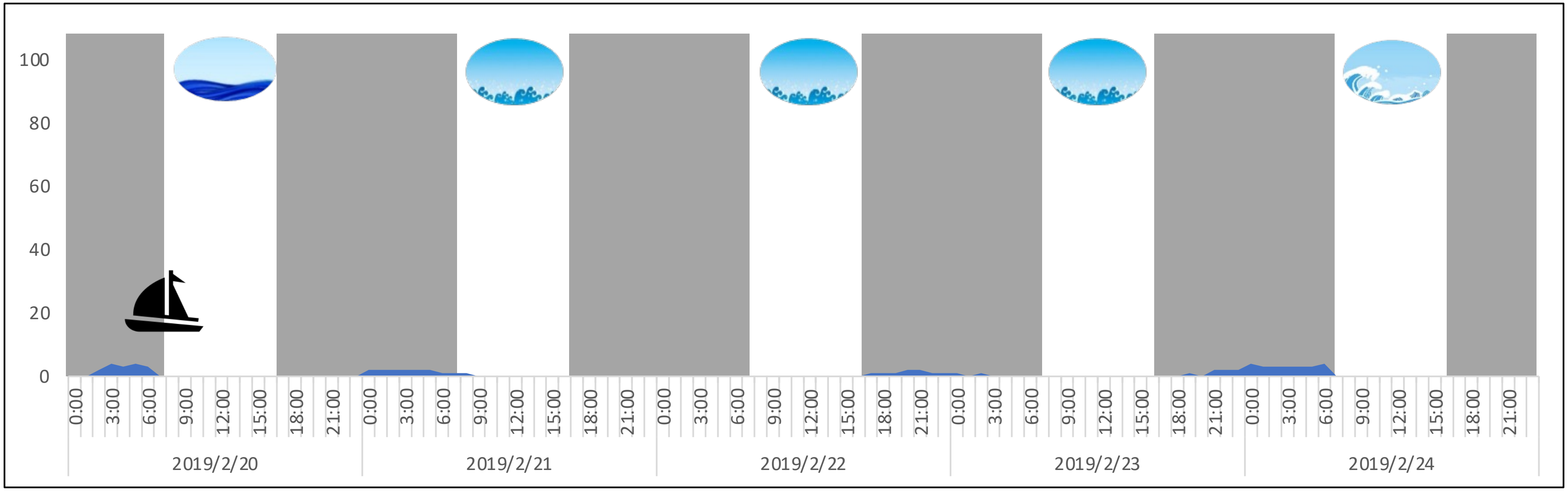
100
80
60
40
20
0
0:00 3:00 6:00 9:00 12:00 15:00 18:00 21:00
2019/2/20
2019/2/21
2019/2/22
2019/2/23
2019/2/24

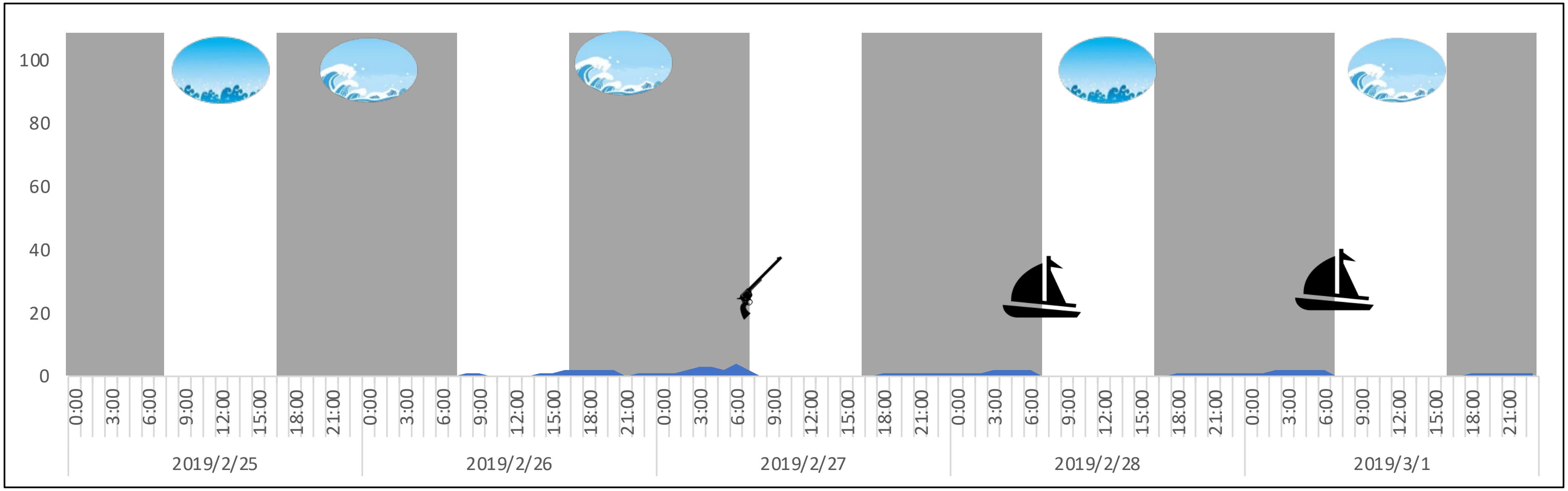
100
80
60
40
20
0
0:00 3:00 6:00 9:00 12:00 15:00 18:00 21:00
2019/2/25
2019/2/26
2019/2/27
2019/2/28
2019/3/1

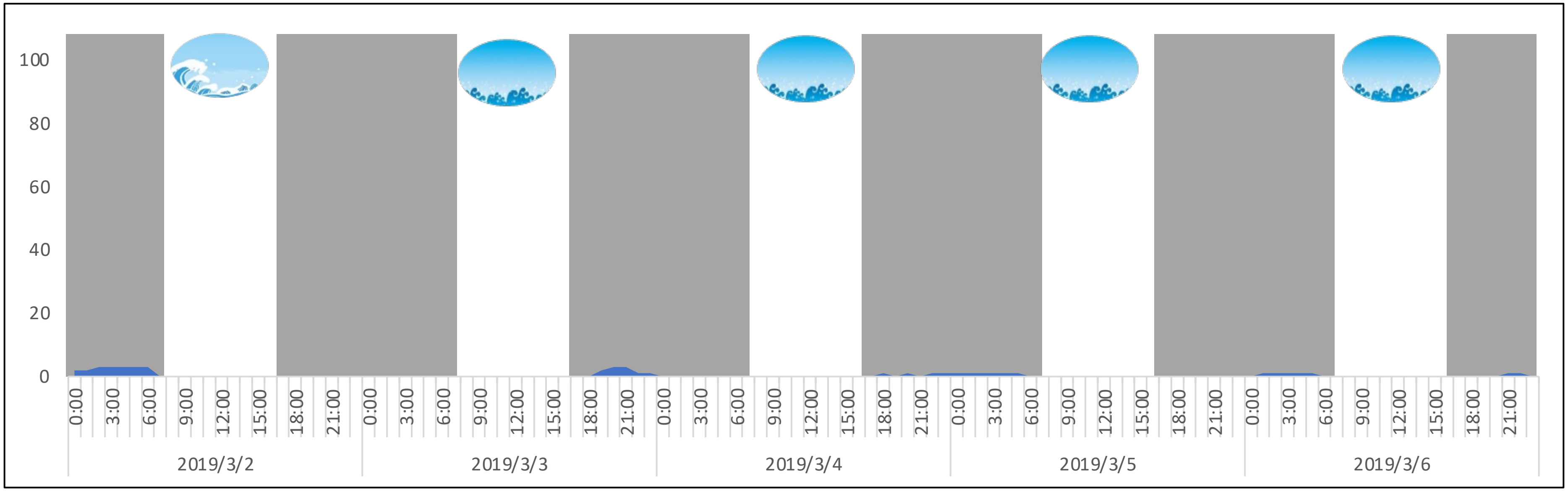

100
80
60
40
20
0
0:00
3:00
6:00
9:00
12:00
15:00
18:00
21:00
2019/3/2
2019/3/3
2019/3/4
2019/3/5
2019/3/6

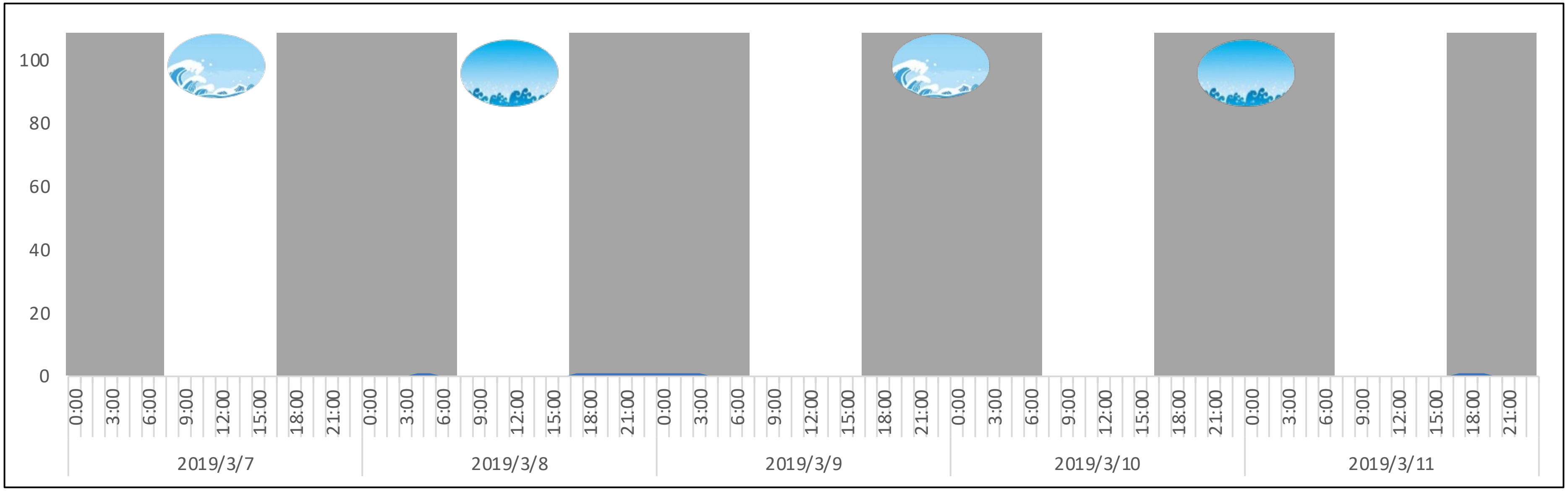

100
80
60
40
20
0
0:00
3:00
6:00
9:00
12:00
15:00
18:00
21:00
2019/3/7
2019/3/8
2019/3/9
2019/3/10
2019/3/11

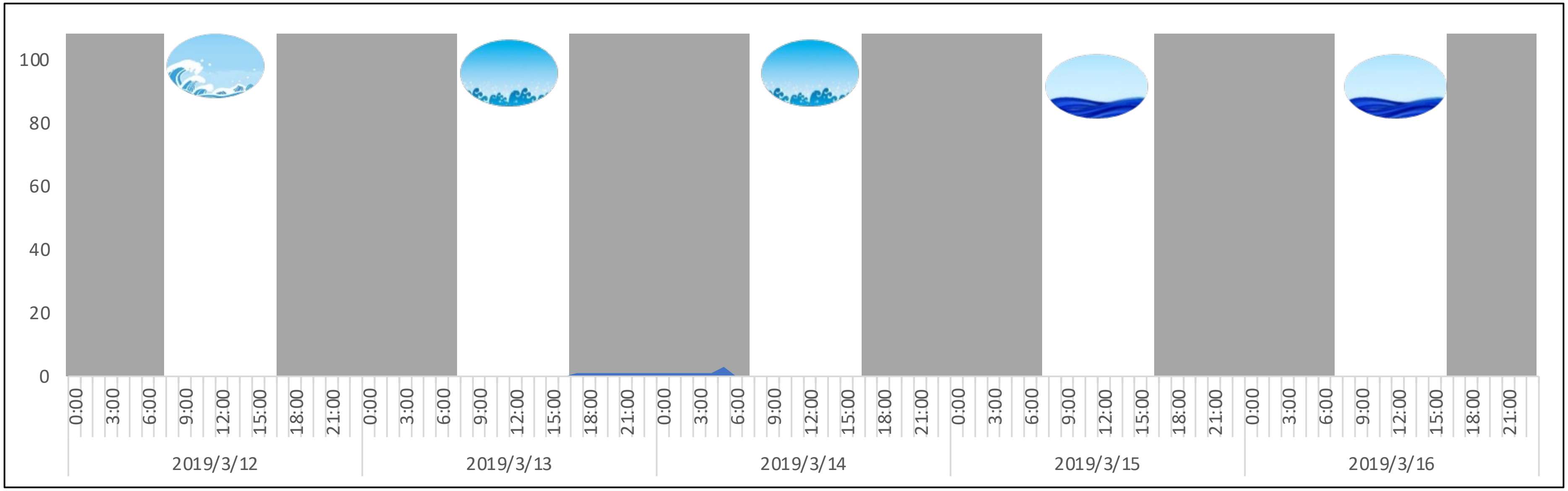

100
80
60
40
20
0
0:00 3:00 6:00 9:00 12:00 15:00 18:00 21:00
2019/3/12
2019/3/13
2019/3/14
2019/3/15
2019/3/16

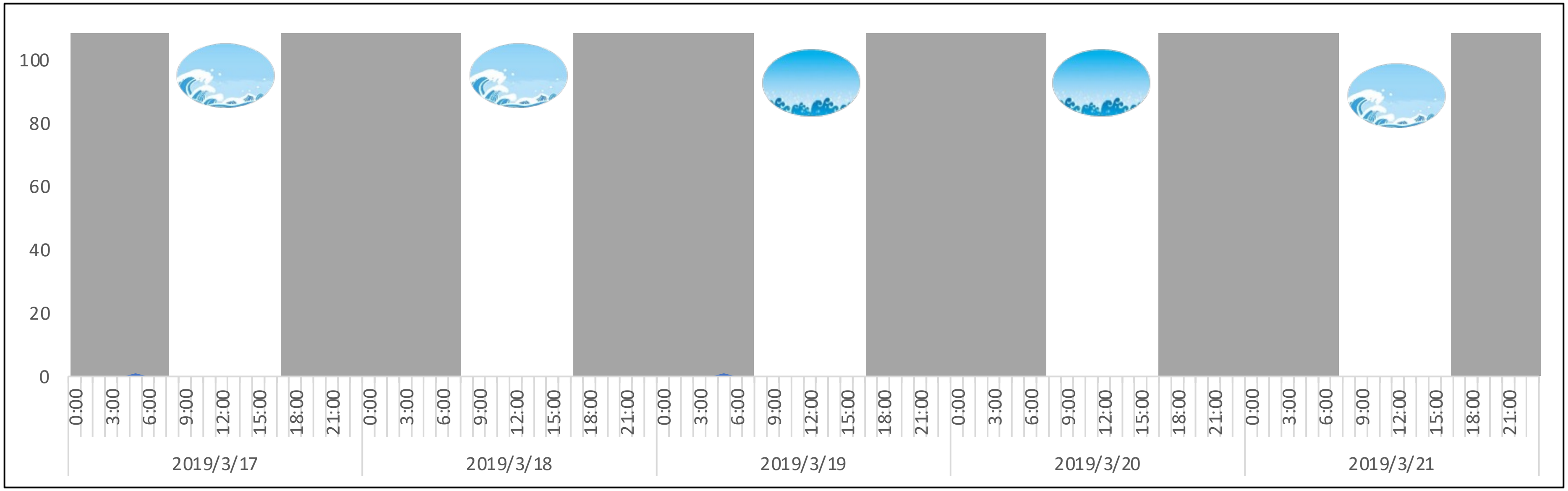

100
80
60
40
20
0
0:00 3:00 6:00 9:00 12:00 15:00 18:00 21:00
2019/3/17
2019/3/18
2019/3/19
2019/3/20
2019/3/21

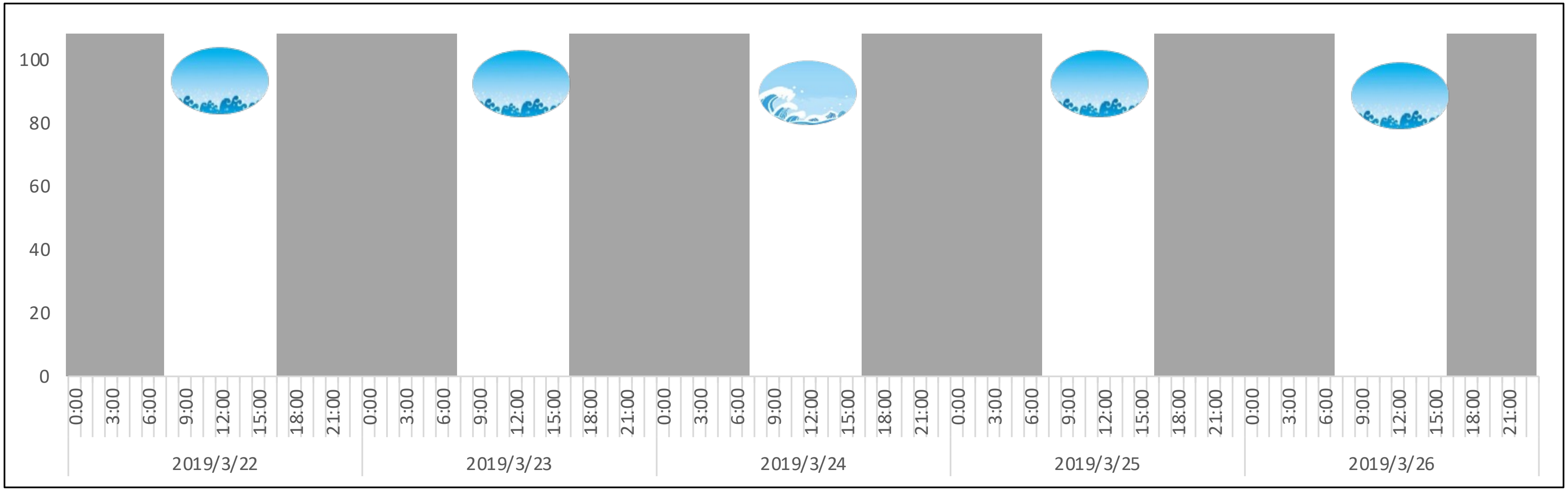

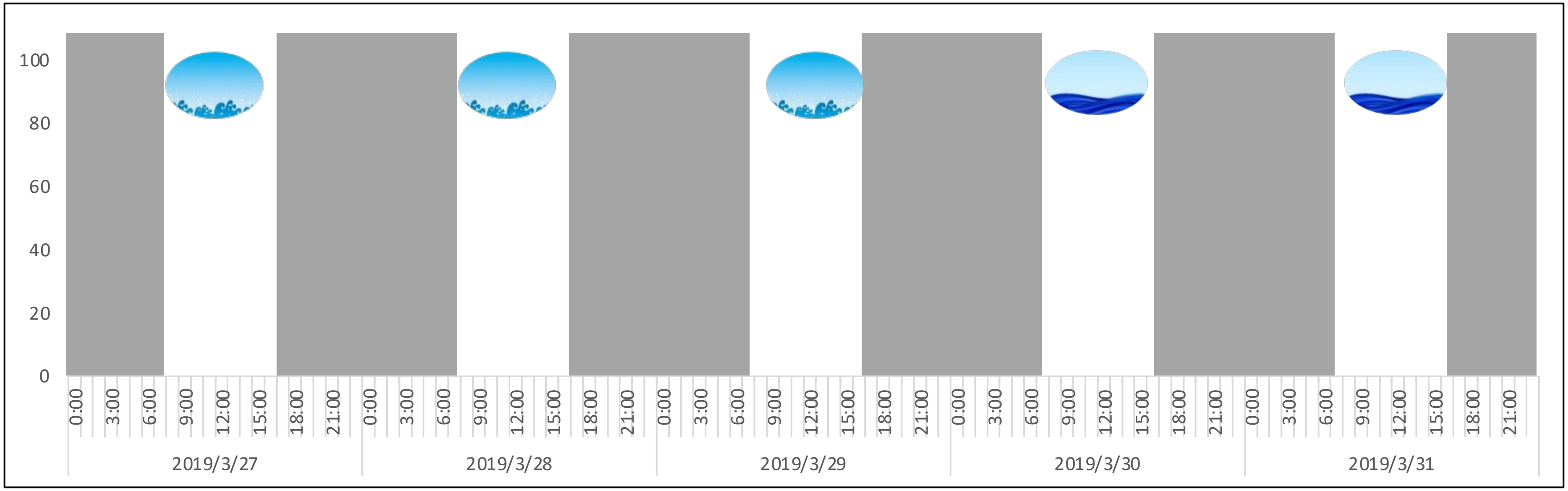

Figure S3. The hourly changes in the number of landed SSLs, weather conditions, and anthropogenic impacts from January 1 to March 31, 2019.
