# Supplementary material for: Human Impact on the Twenty-Four-Hour Patterns of Steller Sea Lions’ Use of a Haulout in Hokkaido, Japan
Source: Animals (Basel). 2024 Apr 27;14(9):1312. doi: 10.3390/ani14091312 (PMC11083395; doi:10.3390/ani14091312)
Supplement: Supplementary file 1 [file animals-14-01312-s001.zip › Table S1.pdf]

Table S1. The annual fishery damages caused by Steller sea lions in the Ishikari area, Shiribeshi area, and whole Hokkaido.

	2009	2010	2011	2012	2013	2014	2015	2016	2017	2018	2019
Hokkaido	1,385,713	1,354,132	1,607,914	1,497,723	1,612,351	1,978,570	1,773,616	1,869,862	1,597,697	1,178,699	1,006,180
Ishikari	296,212	206,221	228,441	211,548	149,522	167,283	201,239	233,392	254,435	187,745	206,663
Shiribeshi	262,705	499,178	490,945	457,089	495,469	628,180	517,175	582,175	399,230	227,354	166,338

The top row indicates years.
The number in the row indicates the amount of fishery damage (Yen in thousands) of each area by Steller sea lions, mainly from January to March
Left column: Hokkaido indicates the whole Hokkaido area; Ishikari indicates the main area where the haulout, “Todo Iwa,” is located; Shiribeshi indicates the next area of Ishikari
